# Light quantum control of persisting Higgs modes in iron-based superconductors

**DOI:** 10.1038/s41467-020-20350-6

**Published:** 2021-01-11

**Authors:** C. Vaswani, J. H. Kang, M. Mootz, L. Luo, X. Yang, C. Sundahl, D. Cheng, C. Huang, R. H. J. Kim, Z. Liu, Y. G. Collantes, E. E. Hellstrom, I. E. Perakis, C. B. Eom, J. Wang

**Affiliations:** 1 Department of Physics and Astronomy, Iowa State University, and Ames Laboratory, Ames, IA 50011 USA; 2 Department of Materials Science and Engineering, University of Wisconsin-Madison, Madison, WI 53706 USA; 3 Department of Physics, University of Alabama at Birmingham, Birmingham, AL 35294-1170 USA; 4 Applied Superconductivity Center, National High Magnetic Field Laboratory, Florida State University, Tallahassee, FL 32310 USA

**Keywords:** Superconducting properties and materials, Terahertz optics, Ultrafast photonics

## Abstract

The Higgs mechanism, i.e., spontaneous symmetry breaking of the quantum vacuum, is a cross-disciplinary principle, universal for understanding dark energy, antimatter and quantum materials, from superconductivity to magnetism. Unlike one-band superconductors (SCs), a conceptually distinct Higgs amplitude mode can arise in multi-band, unconventional superconductors  via strong interband Coulomb interaction, but is yet to be accessed. Here we discover such hybrid Higgs mode and demonstrate its quantum control by light in iron-based high-temperature SCs. Using terahertz (THz) two-pulse coherent spectroscopy, we observe a tunable amplitude mode coherent oscillation of the complex order parameter from coupled lower and upper bands. The nonlinear dependence of the hybrid Higgs mode on the THz driving fields is distinct from any known SC results: we observe a large reversible modulation of resonance strength, yet with a persisting mode frequency. Together with quantum kinetic modeling of a hybrid Higgs mechanism, distinct from charge-density fluctuations and without invoking phonons or disorder, our result provides compelling evidence for a light-controlled coupling between the electron and hole amplitude modes assisted by strong interband quantum entanglement. Such light-control of Higgs hybridization can be extended to probe many-body entanglement and hidden symmetries in other complex systems.

## Abstract (web summary)

A collective excitation called Higgs mode may arise in multi-band superconductors via strong interband interaction, but it is yet to be accessed. Here, the authors observe a tunable coherent amplitude oscillation of the order parameter in Ba(Fe_1−*x*_Co_*x*_)_2_As_2_, suggesting appearance and control of the Higgs mode by light tuning interband interaction.

## Introduction

**Fig. 1:**
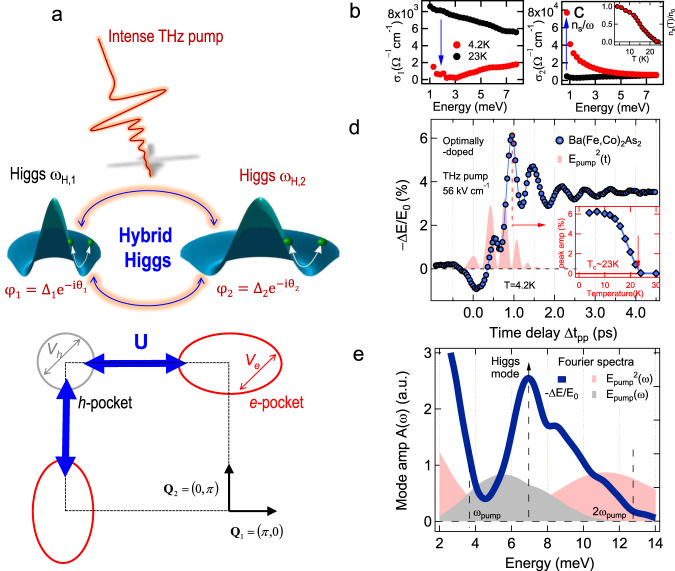
The 2*Δ*_SC_ coherent oscillations detected by two-pulse THz coherent spectroscopy of multi-band FeSCs. **a**  Illustration of coherent excitation of hybrid Higgs mode via THz quantum quench. An effective three-band model has a *h* pocket at the Γ point and two *e* pockets at X/Y points, with strong inter- (blue) and weak intraband (gray and red) interactions marked by arrows. **b**, **c** Real and imaginary parts of the complex THz conductivity spectra *σ*_1_(*ω*) and *σ*_2_(*ω*) in the superconducting (4.2 K) and normal states (23 K) of Ba(Fe_1−*x*_Co_*x*_)_2_As_2_ (*x* = 0.08) in equilibrium. Inset of **c** shows the temperature dependence of the superfluid density normalized to its value at 4.2 K, *n*_*s*_(*T*)/*n*_0_, as determined from 1/*ω* divergence of *σ*_2_(*ω*) (blue arrow in **c**). **d**  Differential THz transmission Δ*E*/*E*_0_ (blue circles) measured by phase-locked two-THz-pulse pump–probe spectroscopy at 4.2 K shows pronounced coherent oscillations for a peak THz field strength of *E*_pump_ = 56 kV/cm. The pink shaded curve denotes the square of the pump THz waveform ${E}_{{\rm{pump}}}^{2}$. Inset: Temperature dependence of the peak amplitude of Δ*E*/*E*_0_. **e**  Fourier spectrum of the coherent oscillations in Δ*E*/*E*_0_ (blue line) exhibits a resonance peak at  ~6.9 meV (blue line) and is distinct from both the pump *E*_pump_ (gray) and pump-squared ${E}_{{\rm{pump}}}^{2}$ (pink) spectra.

Amplitude modes and their competition with charge-density fluctuations are currently intensely studied in one-band superconductors (SCs).  In multi-band, unconventional SCs, a hybrid Higgs amplitude mode, controllable by terahertz (THz) laser pulses, can arise via strong interband Coulomb interaction. A recent prominent example to explore such collective mode is seen in iron-arsenide based superconductors (FeSCs). Phase coherence between multiple SC condensates in different strongly interacting bands is well established in FeSCs. As illustrated in Fig. 1a, a dominant Coulomb coupling between the *h*- and *e*-like Fermi sea pockets, unlike in other SCs, is manifested by, e.g., *s*±  pairing symmetry^1,2^, spin-density wave resonant peaks, and nesting wave vectors (black arrows)^3–6^. Despite the extensive studies, experimental evidence for Higgs amplitude coherent excitations in FeSCs has not been reported yet, despite recent progress in non-equilibrium superconductivity and collective modes^7–22^.

The condensates in different bands of Ba(Fe_1−*x*_Co_*x*_)_2_As_2_ studied here, shown in Fig. 1a, are coupled by the strong interband *e*–*h* interaction *U* (blue double arrow vector), which is about one order of magnitude stronger than the intraband interaction *V* (gray and red double arrows)^23^. For *U* ≫ *V*, the formation channels of collective modes are distinct from one-band SC^7–9,24^ and multi-band MgB_2_ with dominant intraband interaction, *V* ≫ *U*^25,26^. For the latter, only Leggett modes are observed thus far^10^. In contrast, for *U* ≫ *V*, one expects Higgs amplitude modes arising from the condensates in all Coulomb-coupled bands, i.e., in the *h* pocket at the Γ-point (gray circle, mode frequency *ω*_H,1_), and in the two *e* pockets at (0, *π*) and (*π*, 0) (red ellipses, *ω*_H,2_). A single-cycle THz oscillating field (red pulse) can act like a quantum quench, with impulsive non-adiabatic driving of the Mexican-hat-like quantum fields (dark green) and, yet, with minimum heating of other degrees of freedoms. Consequently, the multi-band condensates are forced out of the free energy minima, since they cannot follow the quench adiabatically. Most intriguingly, such coherent nonlinear driving not only excites amplitude mode oscillations in the different Fermi sea pockets, but also transiently modifies their coupling, assisted by the strong interband interaction *U*. Such coherent transient coupling can be regarded as nonlinear amplitude mode hybridization with a time-dependent phase coherence. In this way, THz laser fields can manipulate hybrid Higgs emerging collective modes in FeSCs, assisted by the strong interband interaction.

Here we present evidence of hybrid Higgs modes that are excited and controlled by THz-field-driven interband quantum entanglement in a multi-band SC, optimally doped Ba(Fe_1−*x*_Co_*x*_)_2_As_2_, using two phase-locked near-single-cycle THz laser fields. We reveal a striking nonlinear THz field dependence of coherent amplitude mode oscillations: quick increase to maximum spectral weight (SW) with negligible mode frequency shift, followed by a huge SW reduction by more than 50%, yet with robust mode frequency position, with less than 10% redshift. These distinguishing features of the observed collective mode are different from any one-band and conventional SC results and predictions so far. Instead, they are consistent with coherent coupling between the *e-* and *h*-like amplitude modes . To support this scenario, we perform quantum kinetic modeling of a hybrid Higgs mechanism without invoking extra disorder or phonons. This simulation identifies the key role of the interband interaction *U* for coherently coupling two amplitude modes and for controlling the SW of the lower Higgs mode observed in the experiment.

## Results

### The amplitude coherent oscillations detected by THz two-pulse coherent spectroscopy

The equilibrium complex conductivity spectra, i.e., real and imaginary parts, *σ*_1_(*ω*) and *σ*_2_(*ω*), of our epitaxial Ba(Fe_1−*x*_Co_*x*_)_2_As_2_ (*x* = 0.08) film^27^ (Methods) measure the low-frequency quasi-particle (QP) electrodynamics and condensate coherence, respectively (Fig. 1b, c)^28^. The normal state (black circles) displays Drude-like behavior, while the QP spectral weight in *σ*_1_(*ω*) is depleted in the SC state due to SC gap openings, seen, e.g., in the 4.2 K trace (red circles). The lowest SC gap value 2Δ_1_ ~ 6.8 meV obtained is in agreement with the literature values 6.2–7 meV^29,30^ (Methods). Such *σ*_1_(*ω*) spectral weight depletion is accompanied by an increase of condensate fraction *n*_s_/*n*_0_ (inset, Fig. 1c), extracted from a diverging 1/*ω* condensate inductive response, marked by blue arrow, e.g., in the 4.2 K lineshape of *σ*_2_(*ω*) (Fig. 1c). Note that the superfluid density *n*_s_ vanishes above *T*_c_ ~ 23  K (inset Fig. 1c).

We characterize the THz quantum quench coherent dynamics directly in the time domain^31–33^ (Methods) by measuring the responses to two phase-locked THz pulses as differential field transmission of the weak THz probe field Δ*E*/*E*_0_ (blue circles, Fig. 1d) for THz pump field, *E*_pump_  =  56 kV/cm and as a function of pump–probe time delay Δ*t*_pp_. The central pump energy *ℏω*_pump_  =  5.4 meV (gray shade, Fig. 1e) is chosen slightly below the 2Δ_1_ gap. Intriguingly, the Δ*E*/*E*_0_ dynamics reveals a pronounced coherent oscillation, superimposed on the overall amplitude change, which persists much longer than the THz photoexcitation (pink shade). This mode is excited by the quadratic coupling of the pump vector potential, ${{\bf{A}}}^{2}(t)\propto {E}_{{\rm{pump}}}^{2}/{\omega }^{2}$ due to the SC equilibrium symmetry^7^. Such coherent responses yield information within the general framework of coherent nonlinear spectroscopy^34^ (Methods). The origin of the observed coherent Δ*E*/*E*_0_ oscillation is better illustrated by its Fourier transformation (FT), shown in Fig. 1e. The FT spectrum of the coherent nonlinear signals (blue solid line) displays a pronounced resonance at 6.9 meV, indicative of 2Δ_1_ coherent amplitude mode oscillations. This FT spectrum strongly differs from the spectra of both THz pump *E*_pump_(*ω*) centered at *ω*_pump_ ~ 5.4 meV (gray shade) and second harmonic, Anderson pseudo-spin (APS) precession at 2*ω*_pump_ from ${E}_{{\rm{pump}}}^{2}(\omega )$ (pink shade). The broadband spectrum of the few-cycle pump pulse used in the experiment overlaps with the mode resonances such that the Δ*E*/*E*_0_ oscillates with the collective mode frequencies^9^. After the oscillation, time-dependent complex conductivity spectra, *σ*_1_(*ω*, Δ*t*_pp_) and *σ*_2_(*ω*, Δ*t*_pp_), can be measured (Supplementary Figs. 5–6). They show that Δ*E*/*E*_0_ closely follows the pump-induced change in condensate density, Δ*n*_s_/*n*_0_^13^. The THz excitation at *E*_pump_  =  56 kV/cm only reduces *n*_s_ slightly, Δ*n*_s_/*n*_0_ ~ Δ*E*/*E*_0_ ~ −3% at Δ*t*_pp_  =  5 ps. Furthermore, the pump-induced peak amplitude (blue diamond), marked by the red dashed line in Fig. 1d, diminishes above *T*_c_ (inset). These evidence indicate that the measured coherent oscillations reflect the emergence of a hybrid Higgs multi-band collective mode between two Coulomb-coupled lower and higher modes, *ω*_H,1_ and *ω*_H,2_.

**Fig. 2:**
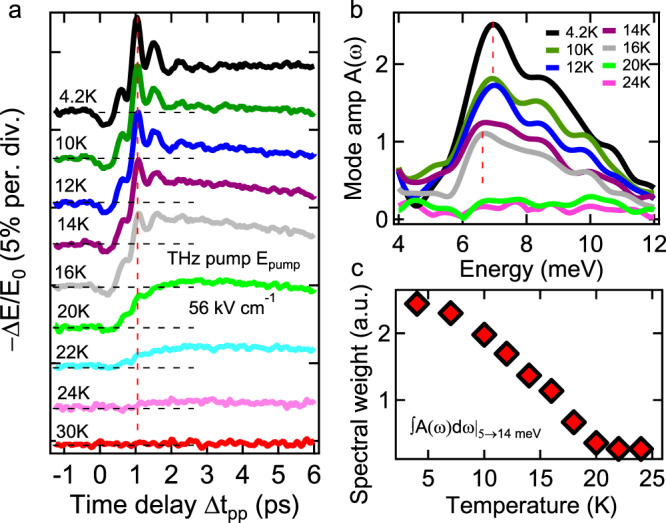
Temperature dependence of hybrid Higgs mode coherent oscillations in FeSCs. **a**  Temporal profiles of  −Δ*E*/*E*_0_ at various temperatures and for a peak THz pump E-field of *E*_pump_ = 56 kV/cm. Traces are offset for clarity. **b**  Fourier spectra of the Higgs mode oscillations derived from the two-pulse coherent pump–probe signals at different temperatures. Dashed red lines indicate the slight redshift of the Higgs mode frequency with the drastic reduction of mode SW as a function of temperature. **c**  Temperature dependence of the integrated SW_5→14meV_ of the Higgs mode in **b**.

Figure 2 reveals a strong temperature dependence of the Higgs mode oscillations. The coherent dynamics of Δ*E*/*E*_0_ is shown in Fig. 2a for temperatures 4.2–30 K. Approaching *T*_c_ from below, the coherent oscillations quickly diminish, as seen by comparing the 4.2 K (black line) and 16 K (gray) traces versus 22 K (cyan) and 24 K (pink) traces. Figure 2b shows the temperature-dependent Fourier spectra of Δ*E*/*E*_0_, in the range 4–12 meV. Figure 2c plots the integrated spectral weight SW_5→14meV_ of the amplitude mode (Fig. 2b). The strong temperature dependence correlates the mode with SC coherence. Importantly, while the mode frequency is only slightly red-shifted, by less than 10% before full SW depletion close to *T*_c_, SW is strongly suppressed, by  ~55% at 16 K (*T*/*T*_c_ ~ 0.7). Such a spectral weight reduction in FeSCs is much larger than expected in one-band BCS superconductors or for *U* = 0 shown later. We also note that the temperature dependence of Higgs oscillations, observed in FeSCs here, has not been measured experimentally in conventional BCS systems, and could represent a key signature of quantum quench dynamics of unconventional SCs. The observed behavior is consistent with our simulations of the hybrid Higgs mode in multi-band SCs with dominant interband *U*, shown later.

### Nonlinear THz field dependence of hybrid Higgs mode in FeSCs

**Fig. 3:**
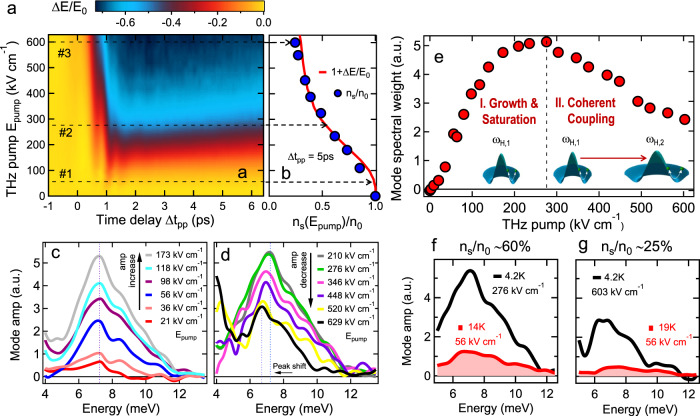
Nonlinear THz field dependence of hybrid Higgs mode in FeSCs. **a**  A 2D false-color plot of Δ*E*/*E*_0_ as a function of pump E-field strength *E*_pump_ and pump–probe delay Δ*t*_pp_ at 4.2 K. **b**  THz pump E-field *E*_pump_ dependence of 1 + Δ*E*/*E*_0_ (red line) overlaid with the superfluid density fraction *n*_*s*_/*n*_0_ (blue circles) after THz pump at Δ*t*_pp_ = 5 ps and *T* = 4.2 K. Dashed arrows (black) mark the three pump *E*-field regimes, i.e., weak, partial, and saturation, identified in the main text. **c**, **d** Spectra of coherent Higgs mode oscillations show a distinct non-monotonic dependence as a function of THz pump field, i.e., a rapid increase in the mode amplitude for low pump E-field strengths up to 173 kV/cm, saturation up to 276 kV/cm and significant reduction at higher fields. The blue dashed line marks the resonance of the mode and the redshift of the Higgs mode peak, much smaller than the mode SW change. **e**  Integrated spectral weight SW_5→14meV_ of the Higgs mode at various pump *E*-field strengths, indicating the SW reduction of the Higgs mode from dominant *ω*_H,1_ at low driving fields to *ω*_H,2_ due to the interband interaction and coherent coupling, illustrated in the inset, at high driving fields above *E*_pump_ = 276 kV/cm. **f**, **g** Contrasting the thermal and THz-driven states of coherent hybrid Higgs mode spectral weight by comparing the mode spectra at the similar superfluid density **f** *n*_*s*_/*n*_0_  =  60% and **g** *n*_*s*_/*n*_0_  =  25% achieved by THz pump (black solid lines) and temperature (red shades).

Figure 3 presents distinguishing experimental evidence for the hybrid Higgs mode in FeSCs, which is different from one-band SCs – a highly nonlinear THz electric field dependence of coherent 2Δ_1_ oscillations that manifests as a huge SW change, yet with persisting mode frequency, i.e., with only very small redshift. Figure 3a shows the detailed pump-fluence dependence of Δ*E*/*E*_0_ as a function of time delay, presented as a false-color plot at *T* = 4.2  K for up to *E*_pump_ ~ 600 kV/cm. It is clearly seen that amplitude mode oscillations depend nonlinearly on the *E*_pump_ field strength. 1 + Δ*E*/*E*_0_ (red solid line) at Δ*t*_pp_  = 5 ps is shown in Fig. 3b. This, together with the measured  ~1/*ω* divergence in *σ*_2_(*ω*, Δ*t*_pp_), allows the determination of the condensate fraction *n*_s_(*E*_pump_)/*n*_0_ (blue circles) in the driven state (Supplementary Fig. 6). There shows three different excitation regimes, marked by black dash lines in Fig. 3a–b: (1) in regime #1, the condensate quench is minimal, e.g., *n*_s_/*n*_0_ ≥ 97% below the field *E*_*#*1_  =  56 kV/cm; (2) Regime #2 displays partial SC quench, where *n*_s_/*n*_0_ is still significant, e.g., condensate fraction  ≈60% at *E*_*#*2_  =  276 kV/cm; (3) A saturation regime #3 is observed  ~*E*_*#*3_  = 600 kV/cm, which leads to a slowly changing *n*_s_/*n*_0_ approaching a saturation ~25%. The saturation is expected since below gap THz pump is used, especially *ℏω*_pump_ ≪ 2Δ_2_ ~ 15–19 meV at the *e-like* pockets^29,30^.

Quantum quenching of the single-band BCS pairing interaction has been well established to induce Higgs oscillations with amplitude scaling as 1/$\sqrt{{\Delta }_{\text{SC},\infty }}$, determined by the long-time asymptotic nonthermal order parameter Δ_SC,*∞*_. The latter decreases with pump field^35–37^. Both model and experimental results establish that the Higgs mode amplitude increases with THz pumping until full depletion of the condensate, concurrent with a continuous Higgs resonance redshift to zero^11,35–37^. In contrast to this expected behavior for conventional SCs, Fig. 3a and the Fourier spectra of the coherent oscillations, Fig. 3c, d, show a distinct, non-monotonic pump-field dependence of the Higgs mode amplitude that is unique here. Specifically, the Fourier spectra exhibit a clear resonance at low pump fluences, which coincides with the frequency of the lower Higgs mode *ω*_H,1_. This resonance grows quickly up to a field of *E*_pump_  =  173 kV/cm (Fig. 3c), saturates up to 276 kV/cm (Fig. 3d) and then exhibits a significant reduction in pump regime #3, e.g., by more than 50% at 629 kV/cm (black line, Fig. 3d). Therefore, the coherent oscillations in Fig. 3a quickly increase in pump regime #1 and saturate in regime #2, prior to any significant mode resonance redshift (blue dashed line, Fig. 3c). Above this relatively low field regime, the oscillation amplitude starts to decrease at 276 kV/cm, even though there is still more than 60% of condensate, marked in Fig. 3b: the driven state is still far from full SC depletion. This striking SW reduction is also seen in the integrated spectral weight analysis, SW_5→14meV_, summarized in Fig. 3e. Most intriguingly, while there is a large reduction of the Higgs mode SW ~ 50% at 629 kV/cm (regime #3), the resonance peak position is nearly persistent, with ≤10% redshift (blue dashed lines, Fig. 3d). These observations of the hybrid Higgs mode differ from any known behavior in one-band SCs, but are consistent with expectations from light-induced nonlinear coherent coupling of Higgs modes in multi-band SCs by a dominant interband interaction *U*.

The distinct mode amplitude and position variation with pump field extracted from the coherent oscillations clearly show the transition from SW growth and saturation to reduction, marked by the black dashed line at *E*_pump_  =  276 kV/cm (Fig. 3e). The saturation and reduction of SW in the amplitude oscillation, yet with a persisting mode frequency, cannot be explained by any known mechanism. This can arise from the coupling of the two amplitude modes *ω*_H,1_ and *ω*_H,2_ expected in iron pnictides due to the strong inter-pocket interaction *U*. The coherent coupling process can be controlled and detected nonlinearly by THz two-pulse coherent spectroscopy, as clearly shown in Fig. 3e. We argue that (I) At low driving fields, *ω*_H,1_ dominates the hybrid collective mode due to less damping than *ω*_H,2_ arising from the asymmetry between the electron and hole pockets; (II) For higher fields, SW of *ω*_H,1_ mode decreases due to the coherent transfer to *ω*_H,2_ mode expected for the strong interband interaction in iron pnictides. Moreover, it is critical to note that the THz driving is of highly nonthermal nature, which is distinctly different from that obtained by temperature tuning in Fig. 2. Specifically, Fig. 3f, g compare the hybrid Higgs mode spectra for similar condensate faction *n*_s_/*n*_0_, i.e., ≈60% (f) and 25% (g), induced by tuning either the temperature (red shade) or THz pump (black line). The mode amplitude is clearly much stronger in the THz-driven coherent states than in the temperature tuned ones.

### Quantum kinetic calculation of the THz-driven hybrid Higgs dynamics

To put the above hybrid Higgs mode findings on a rigorous footing, we calculate the THz coherent nonlinear spectra (Methods) by extending the gauge-invariant density matrix equations of motion theory of ref. ^38^, as outlined in the Supplementary Note 6. Using the results of these calculations, we propose a physical mechanism that explains the distinct differences of the Higgs mode resonance in the four-wave mixing spectra between the strong and weak interband interaction limits. For this, we calculate the APS and quantum transport nonlinearities^38^ driven by two intense phase-locked THz E-field pulses for an effective 3-pocket BCS model of FeSCs^39^. This model includes both intraband and interband pairing interactions, as well as asymmetry between electron and hole pockets. We thus calculate the nonlinear differential field transmission Δ*E*/*E*_0_ for two phase-locked THz pulses, which allows for a direct comparison of our theory with the experiment (Supplementary Note 6).

**Fig. 4:**
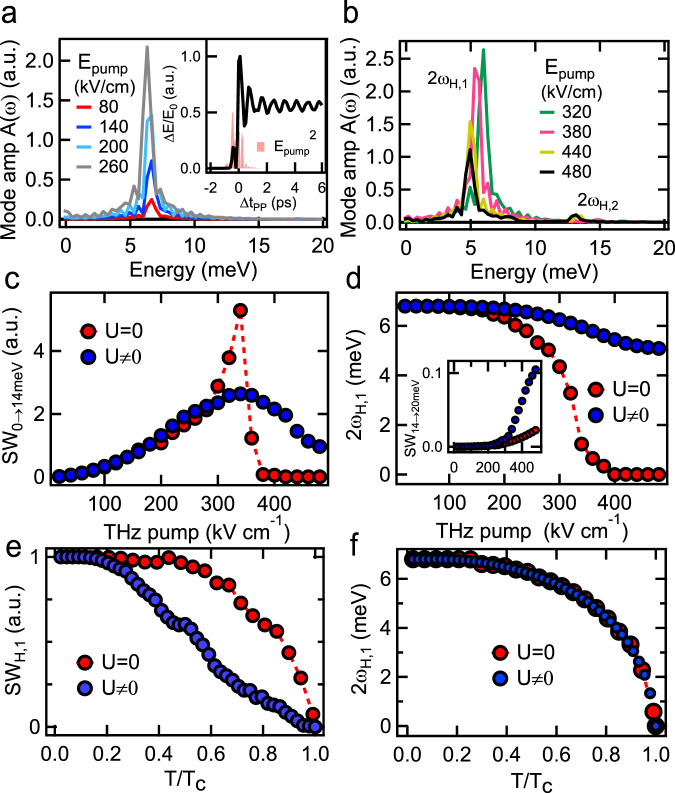
Gauge-invariant quantum kinetic calculation of the THz-driven hybrid Higgs dynamics. **a**  Calculated Higgs mode spectra for low pump E-field strengths. Inset: Calculated Δ*E*/*E*_0_ for peak *E*_pump_  =  380 kV/cm (black) and its comparison with the waveform ${E}_{{\rm{pump}}}^{2}$ (pink, shaded) of the applied THz pump pulse in the experiment. **b**  Calculated Higgs mode spectra for higher field strengths show a decrease in amplitude and redshift of the Higgs mode *ω*_H,1_, consistent with experimentally measured coherent responses in Fig. 3c, d. Note that a second *ω*_H,2_ appears at higher *E*_pump_-field strengths and gets stronger at elevated *E*_pump_-field strengths. Although the *ω*_H,2_ mode is outside the experimental sampling width, it is revealed by the distinct nonlinear THz field dependence of spectral weight controlled by THz pump (Fig. 3d). **c** *E*_pump_-field dependence of the calculated Higgs mode *ω*_H,1_ spectral weight without interband coupling, i.e., *U*  =  0 (red circles) and with strong interband coupling *U*  ≠  0 (blue circles). **d**  Plot of the Higgs mode frequency 2*ω*_H,1_ as a function of *E*_pump_-field strength without interband coupling (red circles) and for strong interband coupling (blue circles). Our simulations for strong *U* (blue circles) are in full agreement with the experimental results in Fig. 3c-d and in sharp contrast with the one-band SC results obtained for *U*  =  0 (red circles). **e**, **f** The calculated spectral weight SW_0→14meV_ **e** and resonance position **f** are plotted as a function of temperature for a fixed pump field of 56.0 kV/cm for *U*  =  0 (red circles) and *U*  ≠  0 (blue circles). With interband coupling, the SW is strongly suppressed, by about 60% up to a temperature of 0.6*T*_c_, while at the same time the mode frequency is only slightly redshifted, by about 15%, before a full spectral weight depletion is observable towards *T*_c_. These simulations are in agreement with the hybrid Higgs behavior in FeSCs and differ from one-band superconductors showing comparable change of SW and position of the Higgs mode with increasing temperature (red circles).

The inset of Fig. 4a presents the calculated Δ*E*/*E*_0_ (black line), shown together with ${E}_{{\rm{pump}}}^{2}(t)$ of the applied experimental pump pulse (pink shade). The calculated Higgs mode spectra, Fig. 4a (regime I) and Fig. 4b (regime II), are dominated by a resonance close to 6.8 meV for low pump fluences, which corresponds to *ω*_H,1_. This resonance grows up to pump fields *E*_THz_ ≈ 320 kV/cm for the parameters used here, with minimal redshift and without any significant SW at *ω*_H,2_ (Fig. 4a). Interestingly, for higher fields (Fig. 4b), we obtain both a redshift and a decrease of the oscillation amplitude. In this regime II, SW emerges close to 15.0 meV, outside of our experimental bandwidth, in the frequency regime of the *ω*_H,2_ Higgs mode. The latter mode is strongly suppressed due to damping induced via electron–hole asymmetry (Supplementary Note 6). Specifically, the ellipticity of the e pockets increases the DOS along the pump field direction and thus increases the damping of mainly the *ω*_H,2_ resonance, which leads to a transfer of oscillator strength to the continuum. This damping has a much smaller influence on the *ω*_H,1_ resonance that arises largely from the hole pockets. Most importantly, the strong interband coupling expected in FeSCs leads to a decrease in the *ω*_H,1_ resonance amplitude, with SW reduction accompanied by a persisting mode frequency. This behavior of the multiband model with strong *U* is clearly seen in the raw experimental data. Note that, while in regime I we observe an increase in the mode amplitude without any significant redshift, in regime II, the decrease in *ω*_H,1_ resonance is accompanied by a small redshift. This behavior of the hybrid Higgs mode contradicts the one-band behavior, recovered by setting *U*  =  0, and is in excellent agreement with our experimental observations in the FeSC system (Fig. 3c–e).

To scrutinize further the critical role of the strong interband interaction *U*, we show the fluence dependence of the coherent Higgs SW close to *ω*_H,1_ in Fig. 4c. SW_0→14meV_ differs markedly between the calculation with strong *U*  ≠  0 (blue circles) and that without inter-pocket interaction *U*  =  0 (red circles), which resembles the one-band BCS quench results. Importantly, the Higgs mode SW_0→14meV_ for strong *U* grows at low pump fluences (regime I), followed by a saturation and then decrease at elevated *E*_pump_ (regime II), consistent with the experiment. Meanwhile, Fig. 4d demonstrates that the resonance frequency remains constant in regime I, despite the strong increase of SW, and then redshifts in regime II, yet by much less than in the one-band system (compare *U*  ≠  0 (blue circles) vs. *U*  =  0 (red circles)). Without inter-pocket *U* (*U*  =  0, red circles in Fig. 4c–d), the SW of Higgs mode *ω*_H,1_ grows monotonically up to a quench of roughly 90%. A further increase of the pump field leads to a complete quench of the order parameter Δ_1_ and a decrease of the SW of Higgs mode *ω*_H,1_ to zero, due to transition from a damped oscillating Higgs phase to an exponential decay. Based on the calculations in Fig. 4c–d, the decrease of the spectral weight with interband coupling appears at a Δ_1_ quench close to 15%, while without interband coupling the decrease of spectral weight is only observable close to the complete quench (~90%) of the SC order parameter Δ_1_. We conclude from this that the spectral weight decrease at the lower Higgs resonance with low redshift is a direct consequence of the strong coupling between the electron and hole pockets due to large *U*. This *E*_pump_ dependence is the hallmark signature of the Higgs mode in FeSCs and is fully consistent with the Higgs mode behaviors observed experimentally.

Finally, the temperature dependence of the hybrid Higgs mode predicted by our model is shown in Fig. 4e, f for *U*  =  0 (red circles) and *U*  ≠  0 (blue circles). With interband coupling, the SW is strongly suppressed, by about 60% up to a temperature of 0.6*T*_c_, while at the same time the mode frequency is only slightly redshifted, by about 15%, before a full spectral weight depletion is observable towards *T*_c_. The strong suppression results from transfer of SW from mode *ω*_H,1_ to the higher mode *ω*_H,2_ with increasing temperature, since the higher SC gap Δ_2_ experiences stronger excitation by the applied pump *E*^2^ with growing *T*. These simulations are in agreement with the hybrid Higgs behavior in Fig. 2 and differ from one-band superconductors showing comparable change of both SW and position of the Higgs mode with increasing temperature (red circles, Fig. 4e, f). Moreover, our calculation without light-induced changes in the collective effects (only charge-density fluctuations) produces a significantly smaller Δ*E*/*E*_0_ signal in the non-perturbative excitation regime (Supplementary Fig. 11). Therefore, we conclude that the hybrid Higgs mode dominates over charge-density fluctuations in two-pulse coherent nonlinear signals in FeSCs, due to the different effects of the strong interband *U* and multi-pocket bandstructure on QPs and on Higgs collective modes.

In summary, we provide distinguishing features for hybrid Higgs modes and thier coherent excitations in multi-band FeSCs, which differ significantly from any previously observed collective mode in other superconducting materials: 2Δ_SC_ amplitude oscillations displaying a robust mode resonance frequency position despite a large change of its spectral weight, more than 50%, on the THz electric field. This unusual nonlinear quantum behavior provides evidence for Higgs hybridization from the interband quantum entanglement in FeSCs. Such discovery and light-control of the hybrid Higgs mode  in multi-band unconventional SCs inspire future research of quantum tomography of  many-body correlated states in complex systems.

## Methods

### Sample preparation

We measure optimally Co-doped BaFe_2_As_2_ epitaxial single-crystal thin films^27^ which are discussed in Supplementary Figs. 1 and 2. They are 60 nm thick, grown on 40 nm thick SrTiO_3_ buffered (001)-oriented (La;Sr)(Al;Ta)O_3_ (LSAT) single-crystal substrates. The sample exhibits a SC transition at *T*_c_ ~ 23  K (Supplementary Fig. 2). The base pressure is below 3 ×  10^−5^ Pa and the films were synthesized by pulsed laser deposition with a KrF (248 nm) ultraviolet excimer laser in a vacuum of 3  ×  10^−4^ Pa at 730^∘^C (growth rate: 2.4 nm/sec). The Co-doped Ba-122 target was prepared by solid-state reaction with a nominal composition of Ba/Fe/Co/As = 1:1.84:0.16:2.2. The chemical composition of the thin film is found to be Ba(Fe_0.92,_Co_0.08_)_2_As_1.8_, which is close to the stoichiometry of Ba122 with 8% (atomic %) optimal Co-doping. The PLD targets were made in the same way using the same nominal composition of Ba(Fe_0.92,_Co_0.08_)_2_As_2.2_ (Supplementary Note 1). The epitaxial and crystalline quality of the optimally doped BaFe_2_As_2_ thin films was measured by four-circle X-ray diffraction (XRD) shown in Supplementary Fig. 1 for the out-of-plane *θ*–2*θ* scans of the films. As an example, the full width half maximum (FWHM) of the (004) reflection rocking curve of the films is as narrow as 0.7^∘^, which indicates high-quality epitaxial thin films. Furthermore, we have also performed chemical, structural, and electrical characterizations of epitaxial Co-doped BaFe_2_As_2_ (Ba-122) superconducting thin films. We determined the chemical composition of Ba122 thin films by wavelength dispersive spectroscopy (WDS) analyses. The chemical composition of the thin film is found to be Ba(Fe_0.92,_Co_0.08_)_2_As_1.8_, which is close to the stoichiometry of Ba122 with 8% (atomic %) optimal Co-doping. We measured temperature-dependent electrical resistivity for superconducting transitions by four-point method (Supplementary Fig. 2). Onset *T*_c_ and *T*_c_ at zero resistivity are as high as 23.4 and 22.0 K, respectively, and Δ*T*_c_ is as narrow as 1.4 K, which are the highest and narrowest values for Ba-122 thin films. In our prior papers, one can also check a zero-field-cooled magnetization *T*_c_ and it clearly shows a diamagnetic signal by superconducting quantum interference device (SQUID) magnetometer measurements.

### Properties of the thin film Co-doped BaFe_2_As_2_ samples used

The lowest SC gap values 2Δ_1_ ~ 6.8 meV were obtained in our THz conductivity data using a Mattis-Bardeen approach similar to that used in^28–30^. Specifically, Supplementary Fig. 7 plots together the THz conductivity of our single-crystal film sample and prior FTIR conductivity of single-crystal samples at the similar doping^29^. They show an excellent agreement which indicate similar superconducting energy gaps of both samples. The two superconducting energy gaps obtained from the FTIR data are 2Δ_1_ ~ 6.2 meV that agrees with our 2Δ_1_ value, and 2Δ_2_ ~ 14.8–19 meV^29,30^. The further analysis of THz conductivity spectra of our sample also allows the determination of other key equilibrium electrodynamics and transport parameters, consistent with the prior literature^3,6^. First, we can obtain the plasma frequency *ω*_*p*_, obtained by fitting the normal state conductivity spectra with the Drude model, which gives the plasma frequency to be   ~194 THz (~804 meV) and the scattering rate  ~2.5 THz (~10 meV). These measurements are in excellent agreement with bulk single-crystal samples at optimal doping^29^: a normal state Drude plasma frequency of 972 meV, scattering rate 15 meV was obtained there. Second, the London penetration depth *λ*_*L*_ = 378 nm, corresponding to a condensed fraction of  ~50%. These are again consistent with the prior measurements of high quality bulk samples, which show ~50% of the free carriers participating in superfluidity below *T*_c_ and a penetration depth of 300 nm.

### Two-pulse THz coherent nonlinear spectroscopy

Our THz pump–THz probe setup, illustrated in Supplementary Figs. 3 and 4, can be understood within the general framework of THz 2D spectroscopy. The experiment is driven by a 1 kHz fs laser amplifier^40^ and performed in the collinear geometry with two pulses *E*_A_ and *E*_B_ with wave vectors ${\overrightarrow{k}}_{A}$=${\overrightarrow{k}}_{B}$=${\overrightarrow{k}}_{{\rm{NL}}}$. Measuring the electric fields in time domain through electro-optic sampling (EOS) by a third pulse (red shade) allows for phase-resolved detection of the sample response. In general, such a two pulse experiment can provide a number of nonlinear (NL) responses as shown in the Supplementary Table 1. The signals arise from the third order (*χ*^(3)^) nonlinear pump–probe responses of the superconducting state, which are separated from the linear response background. Two main contributions relevant here are measured along the same phase matching direction ${\overrightarrow{k}}_{{\rm{NL}}}$: (1) pump–probe (PP) signals that access condensate quench and recovery; (2) Four-wave mixing (FWM) signals that access amplitude channel coherence and/or density fluctuations. For the sake of consistency, we follow our prior publications^13^ to label the time delays Δ*t*_pp_ and *t*_gate_ corresponding to pulse *E*_A_ and *E*_B_ in Supplementary Fig. 3, which can be varied independently. For the PP contribution, a polarization response $\widetilde{P}(\omega ,\Delta {t}_{\text{pp}})$ is measured and used to obtain the time- and frequency-resolved response functions by performing deconvolution along the *t*_gate_ axis. For the FWM contribution, one usually measures it in terms of the inter-pulse delay *τ*  =  *t*_gate_  −  Δ*t*_pp_^34^ as Δ*E*_FWM_(*τ*, *t*_gate_)  =  *E*_AB_(*t*_g_  −  Δ*t*_pp_, *t*_gate_)  −  *E*_A_(Δ*t*_pp_)  −  *E*_B_(*t*_gate_) where *E*_AB_ means *E*_A_ and *E*_B_ are both present. In our experiment, two optical choppers synchronized by the *f* = 1 kHz laser repetition rate modulate the pump and probe THz beams at *f*/2 (500 Hz) and *f*/4 (250 Hz), respectively. The measured data is then divided into 4 channels CH0 to CH3 as shown in the schematic of the timing sequence in Supplementary Fig. 4. After the data acquisition in each channel, we obtain different background-free nonlinear signals as Δ*E*  =  *E*_AB_  −  *E*_A_  −  *E*_B_  =  *E*_CH0_  +  *E*_CH1_  −  *E*_CH2_  −  *E*_CH3_. We provide more details in supplementary.

### Time-resolved complex THz conductivity spectra

Unlike for the Fourier spectra of coherent FWM signals of main interest here, THz PP signals (Supplementary Table 1 and Supplementary Eq. (1), Supplementary Note 2) directly access the time-dependent complex conductivity spectra, i.e., real and imaginary parts, *σ*_1_(*ω*, Δ*t*_PP_), *σ*_2_(*ω*, Δ*t*_PP_), shown in Supplementary Fig. 5 for a fixed time delay Δ*t*_PP_ = 5 ps. PP signal measurements extend the conventional, equilibrium complex conductivity *σ*_1_(*ω*) and *σ*_2_(*ω*) measurement (black and gray circles, Supplementary Fig. 5) to characterize the non-equilibrium post-THz-quench superconducting states. *σ*_1_(*ω*, Δ*t*_PP_), *σ*_2_(*ω*, Δ*t*_PP_) are shown for E-field = 44 kV/cm (red circles) and E-field = 88 kV/cm (blue circles) in Supplementary Fig. 5. The static conductivity can be fitted with Drude-like behavior of quasi-particles in the normal state (black circles) and condensate gaps of 2Δ =  6.8 meV in the SC state (red circles) (discussed later in Supplementary Fig. 7). The PP conductivity spectra identify the minimal quenching of condensate density of few percent for tens of kV/cm E-field used in observing coherent oscillations. Time-dependence of superfluid density can be directly obtained by the diverging *σ*_2_(*ω*, Δ*t*_PP_) ∝ *n*_*s*_/*ω* in Supplementary Fig. 5. Furthermore, we also plot Supplementary Fig. 6a from three representative line-cuts in Fig. 3a (main text) and then compare this with the condensate fraction (Supplementary Fig. 6b) extracted from the usual diverging response in *σ*_2_(*ω*) (Supplementary Fig. 6c). It is clear that, after the oscillation, Δ*E*/*E*_0_ closely follows the pump-induced change in condensate density, Δ*n*_s_/*n*_0_. For example, for weak THz excitation *E*_pump_  =  56 kV/cm and below *T*_*c*_, the THz pump pulse only reduces *n*_s_ slightly, e.g., Δ*E*/*E*_0_ ∝ Δ*n*_s_/*n*_0_ ~ −3%.

### Gauge-invariant theory and simulations of THz coherent nonlinear spectroscopy in FeSCs

We start from the microscopic spatial-dependent Boguliobov–de Gennes Hamiltonian for multi-band superconductors

1 $$H 	= \mathop{\sum }\limits_{\nu ,\alpha }\int {{\rm{d}}}^{3}{\bf{x}}\ {\psi }_{\alpha ,\nu }^{\dagger }({\bf{x}})\left[{\xi }_{\nu }({\bf{p}}+e{\bf{A}}({\bf{x}},t))-\mu -e\phi ({\bf{x}},t)+{\mu }_{{\rm{F}}}^{\alpha ,\nu }({\bf{x}})\right]{\psi }_{\alpha ,\nu }({\bf{x}})\\ 	\quad-\mathop{\sum }\limits_{\nu }\int {{\rm{d}}}^{3}{\bf{x}}\left[{\Delta }_{\nu }({\bf{x}}){\psi }_{\uparrow ,\nu }^{\dagger }({\bf{x}}){\psi }_{\downarrow ,\nu }^{\dagger }({\bf{x}})+{\rm{h.c.}}\right].$$

Here the Fermionic field operators ${\psi }_{\alpha ,\nu }^{\dagger }({\bf{x}})$ and *ψ*_*α*,*ν*_(**x**) create and annihilate an electron with spin *α* in pocket *ν*; *ξ*_*ν*_(**p** + *e***A**(**x**, *t*)) corresponds to the dispersion of the pocket with momentum operator **p** = −i∇_**x**_ (*ℏ* = 1), vector potential **A**(**x**, *t*), and electron charge  −*e*; *μ* is the chemical potential while *ϕ*(**x**, *t*) denotes the scalar potential.

The SC complex order parameter components arising from the condensates in the different Fermi sea pockets are

2 $${\Delta }_{\nu }({\bf{x}})=-2\mathop{\sum }\limits_{\lambda }{g}_{\nu ,\lambda }\langle {\psi }_{\downarrow ,\lambda }({\bf{x}}){\psi }_{\uparrow ,\lambda }({\bf{x}})\rangle =| {\Delta }_{\nu }({\bf{x}})| {{\rm{e}}}^{{\rm{i}}{\theta }_{\nu }({\bf{x}})}\ ,$$

while the Fock energy is given by

3 $${\mu }_{{\rm{F}}}^{\alpha ,\nu }({\bf{x}})=-{g}_{\nu ,\nu }{n}_{\alpha ,\nu }({\bf{x}})\ ,\quad {n}_{\alpha ,\nu }({\bf{x}})=\langle {\psi }_{\alpha ,\nu }^{\dagger }({\bf{x}}){\psi }_{\alpha ,\nu }({\bf{x}})\rangle \ ,$$

and ensures charge conservation in the SC system. Here *g*_*λ*,*ν*_ is the effective inter (*λ* ≠ *ν*) and intra (*λ* = *ν*) electron–electron interaction in the BCS theory developed in previous works.

Hamiltonian (1) is gauge-invariant under the gauge transformation

4 $${\Psi }_{\nu }({\bf{x}})\ \to \ {{\rm{e}}}^{{\rm{i}}{\tau }_{3}\Lambda ({\bf{x}})/2}{\Psi }_{\nu }({\bf{x}})$$

when vector potential, scalar potential, and different order parameter component phases transform as

5 $$\begin{array}{l}{\bf{A}}({\bf{x}})\ \to \ {\bf{A}}({\bf{x}})+\frac{1}{2e}\nabla \Lambda ({\bf{x}})\ ,\ \phi ({\bf{x}})\ \to \ \phi ({\bf{x}})\\ \quad-\frac{1}{2e}\frac{\partial }{\partial t}\Lambda ({\bf{x}})\ ,\ {\theta }_{\nu }({\bf{x}})\ \to \ {\theta }_{\nu }({\bf{x}})+\Lambda ({\bf{x}}),\end{array}$$

with the field operator for band *ν* in Nambu space ${\Psi }_{\nu }({\bf{x}})={({\psi }_{\uparrow ,\nu }({\bf{x}}),{\psi }_{\downarrow ,\nu }^{\dagger }({\bf{x}}))}^{T}$ and Pauli spin matrix $${\tau }_{3}=\left(\begin{array}{ll}1&0\\ 0&-1\end{array}\right)$$. The conventional density matrix describing band *ν*, ${\rho }^{(\nu )}({\bf{x}},{\bf{x}}^{\prime} )=\langle {\Psi }_{\nu }{({\bf{x}})}^{\dagger }{\Psi }_{\nu }({\bf{x}}^{\prime} )\rangle$, depends on the choice of the gauge. To simplify the gauge transformation of the density matrix, we define center-of-mass and relative coordinates ${\bf{R}}=({\bf{x}}+{\bf{x}}^{\prime} )/2$ and ${\bf{r}}={\bf{x}}-{\bf{x}}^{\prime}$ and introduce a new density matrix,

6 $$\begin{array}{l}{\tilde{\rho }}^{(\nu )}({\bf{r}},{\bf{R}})=\exp \left[-{\rm{i}}e\mathop{\int}\nolimits_{0}^{\frac{1}{2}}{\rm{d}}\lambda \ {\bf{A}}({\bf{R}}+\lambda \ {\bf{r}},t)\cdot {\bf{r}}\ {\tau }_{3}\right]\\ \quad \times {\rho }^{(\nu )}({\bf{r}},{\bf{R}})\exp \left[-{\rm{i}}e\mathop{\int}\nolimits_{-\frac{1}{2}}^{0}{\rm{d}}\lambda \ {\bf{A}}({\bf{R}}+\lambda \ {\bf{r}},t)\cdot {\bf{r}}\ {\tau }_{3}\right],\end{array}$$

where ${\rho }^{(\nu )}({\bf{r}},{\bf{R}})=\left.\left\langle {\Psi }_{\nu }^{\dagger }\left({\bf{R}}+\frac{{\bf{r}}}{2}\right){\Psi }_{\nu }\left({\bf{R}}-\frac{{\bf{r}}}{2}\right)\right)\right\rangle$. This new density matrix ${\tilde{\rho }}^{(\nu )}({\bf{r}},{\bf{R}})$ transforms as

7 $${\tilde{\rho }}^{(\nu )}({\bf{r}},{\bf{R}})\ \to \ \exp \left[{\rm{i}}{\tau }_{3}\Lambda ({\bf{R}})/2\right]{\tilde{\rho }}^{(\nu )}({\bf{r}},{\bf{R}})\exp \left[-{\rm{i}}{\tau }_{3}\Lambda ({\bf{R}})/2\right]$$

under the gauge transformation (4), where the transformed phase Λ(**R**) only depends on the center-of-mass coordinate and not on both coordinates **R** and **r** as in the original density matrix. This property simplifies the gauge-invariant description of the photo-excited non-equilibrium SC dynamics.

The equation of motion for ${\tilde{\rho }}^{(\nu )}({\bf{r}},{\bf{R}})$ is now derived by using the Heisenberg equation of motion technique. To simplify the equations of motion, we Fourier transform the obtained exact results with respect to the relative coordinate **r** and then apply a gradient expansion which is valid for SC systems with condensate center-of-mass spatial fluctuations smoother than the spatial dependence of Cooper pair relative motion. To simplify the problem further, we eliminate the phase of the order parameter ${\Delta }_{{\nu }_{0}}({\bf{R}})$ by applying the gauge transformation

8 $${\tilde{\rho }}^{(\nu )}({\bf{k}},{\bf{R}})={{\rm{e}}}^{-{\rm{i}}{\tau }_{3}{\theta }_{{\nu }_{0}}({\bf{R}})/2}{\tilde{\rho }}^{(\nu )}({\bf{k}},{\bf{R}}){{\rm{e}}}^{{\rm{i}}{\tau }_{3}{\theta }_{{\nu }_{0}}({\bf{R}})/2}.$$

After assuming a homogeneous SC system and homogeneous excitation conditions by neglecting **R**-dependence, we obtain the gauge-invariant Bloch equations for multi-band superconductors that were solved numerically here

9 $${\rm{i}}\frac{\partial }{\partial t}{\tilde{\rho }}_{1,1}^{(\nu )}({\bf{k}})	= -{\rm{i}}\ e\ {\bf{E}}(t)\cdot {\nabla }_{{\bf{k}}}{\tilde{\rho }}_{1,1}^{(\nu )}({\bf{k}})-| {\Delta }_{\nu }| \\ 	\quad\times \left[{{\rm{e}}}^{{\rm{i}}\delta {\theta }_{\nu }}{\tilde{\rho }}_{1,2}^{(\nu )}({\bf{k}}-{{\bf{p}}}_{{\rm{S}}}/2)-{{\rm{e}}}^{-{\rm{i}}\delta {\theta }_{\nu }}{\tilde{\rho }}_{2,1}^{(\nu )}({\bf{k}}-{{\bf{p}}}_{{\rm{S}}}/2)\right]\ ,\\ {\rm{i}}\frac{\partial }{\partial t}{\tilde{\rho }}_{2,2}^{(\nu )}({\bf{k}})	= \, {\rm{i}}\ e\ {\bf{E}}(t)\cdot {\nabla }_{{\bf{k}}}{\tilde{\rho }}_{2,2}^{(\nu )}({\bf{k}})+| {\Delta }_{\nu }|\\ 	\quad\times \left[{{\rm{e}}}^{{\rm{i}}\delta {\theta }_{\nu }}{\tilde{\rho }}_{1,2}^{(\nu )}({\bf{k}}+{{\bf{p}}}_{{\rm{S}}}/2)-{{\rm{e}}}^{-{\rm{i}}\delta {\theta }_{\nu }}{\tilde{\rho }}_{2,1}({\bf{k}}+{{\bf{p}}}_{{\rm{S}}}/2)\right]\ ,\\ {\rm{i}}\frac{\partial }{\partial t}{\tilde{\rho }}_{1,2}^{(\nu )}({\bf{k}})	= -[{\xi }_{\nu }({\bf{k}}-{{\bf{p}}}_{{\rm{S}}}/2)+{\xi }_{\nu }(-{\bf{k}}-{{\bf{p}}}_{{\rm{S}}}/2)+2({\mu }_{{\rm{eff}}}+{\mu }_{{\rm{F}}}^{\nu })]{\tilde{\rho }}_{1,2}^{(\nu )}({\bf{k}})\\ 	{\,\,\,\,\,\,}+| {\Delta }_{\nu }| {{\rm{e}}}^{-{\rm{i}}\delta {\theta }_{\nu }}\left[{\tilde{\rho }}_{2,2}^{(\nu )}({\bf{k}}-{{\bf{p}}}_{{\rm{S}}}/2)-{\tilde{\rho }}_{1,1}^{(\nu )}({\bf{k}}+{{\bf{p}}}_{{\rm{S}}}/2)\right].$$

Here we introduced the gauge-invariant superfluid momentum

10 $${{\bf{p}}}_{{\rm{S}}}=-2\ e\ {\bf{A}}$$

and effective chemical potential

11 $${\mu }_{{\rm{eff}}}=e\ \phi +\frac{1}{2}\frac{\partial }{\partial t}{\theta }_{{\nu }_{0}}-\mu \ .$$

The Leggett mode corresponds to oscillations of the phase difference

12 $$\delta {\theta }_{\nu }={\theta }_{{\nu }_{0}}-{\theta }_{\nu }\ ,$$

while the Higgs mode is defined by the amplitude oscillation of the multi-component complex SC order parameter. The latter is expressed in terms of the gauge-invariant density matrix (6). To lowest order in the gradient expansion,

13 $$| {\Delta }_{\nu }| =-2{{\rm{e}}}^{-{\rm{i}}\delta {\theta }_{\nu }}\mathop{\sum }\limits_{\lambda }{g}_{\nu ,\lambda }{\tilde{\rho }}_{2,1}^{\lambda }({\bf{k}})\ .$$

There are three mechanisms contributing to the driving of the Higgs modes. First, quantum transport contributions  ∝ **E** in the equations of motion (9) lead to an acceleration of the Cooper pair condensate by the pump electric field (Lightwave Quantum Electronics)

14 $$\frac{\partial }{\partial t}{{\bf{p}}}_{{\rm{S}}}=2e\ {\bf{E}}\ ,$$

which is neglected in the Anderson pseudo-spin model. This results in SC order parameter nonlinearities that are of odd order in the electric field. The condensate acceleration breaks equilibrium-inversion symmetry of the SC system and can lead to dc supercurrent generation when lightwave propagation effects are included. Second, the minimal coupling, *ξ*(**k** − **p**_S_/2) + *ξ*(**k** + **p**_S_/2), known from the Anderson pseudo-spin model drives even-order nonlinearities of the SC order parameter and depends on the band dispersion non-parabolicity. Third, the induced condensate momentum leads to a displacement of populations and coherences in momentum space by **p**_S_/2 in the equations of motion which is also neglected in the Anderson pseudo-spin model. While the linear coupling to the superconductor via the quantum transport terms dominate the driving of the Higgs modes in the perturbative excitation regime, the excitation of the hybrid Higgs mode in the non-perturbative regime is dominated by the quadratic ${{\bf{p}}}_{{\rm{S}}}^{2}$-coupling known from the Anderson pseudo-spin model^38^.

In our calculations we solve the gauge-invariant optical Bloch equations (9) for a 3-pocket model with a hole (h) pocket centered at the Γ-point and two electron (e) pockets located at (*π*, 0) and (0, *π*). We include the inter e–h pocket interactions (*U*  =  *g*_e,h_  =  *g*_h,e_) as well as intra-pocket interactions (*V*_*λ*_  =  *g*_*λ*,*λ*_) while inter e–e pocket interactions are neglected for simplicity. The dominance of interband coupling *U* between e–h pockets over intraband in Fe-based SCs is taken into account by using an interband-to-intraband interaction ratio of *r*  =  *U*/*V*  =  10. The pockets are modeled using the square lattice nearest-neighbor tight-binding dispersion ${\xi }_{\nu }({\bf{k}})=-2\ [{J}_{\nu ,x}\cos ({k}_{x})+{J}_{\nu ,y}\cos ({k}_{y})]+{\mu }_{\nu }$ with hopping parameter *J*_*ν*,*i*_ and band-offset *μ*_*ν*_. We choose a circular hole pocket with *J*_1,*x*_  =  *J*_1,*y*_  =  10.0 meV and *μ*_1_  = −37.5   meV. We introduce the known particle–hole asymmetry between electron and hole pockets in our system by considering elliptical electron pockets with *J*_2,*x*_  =  *J*_3,*y*_  =  −10.0 meV, *J*_2,*y*_  =  *J*_3,*x*_  = −50.0 meV, and *μ*_2_  =  *μ*_3_  =  57.5 meV. The latter can lead to coexistence of superconductivity and spin-density wave by changing the doping level. For the doping levels considered here, such asymmetry in our calculation strongly suppresses the *ω*_H,2_ mode in the spectra of coherent Δ*E*/*E* dynamics presented in Fig. 4a–b in the main text as discussed in more detail below. We assume *s*± -pairing symmetry with equilibrium SC order parameters Δ_1_  =  3.4 meV for the hole pocket and Δ_2_  =  Δ_3_  =  9.7 meV for the electron pockets. To directly model our phase-coherent nonlinear pump–probe spectroscopy experiments analogous to 2D THz phase-coherent nonlinear spectroscopy in semiconductors, we calculate the nonlinear differential transmission, Δ*E*, and not just the order parameter dynamics. The nonlinear differential transmission Δ*E*/*E*_0_ is obtained by computing the transmitted *E*-field of both pump and probe pulse, *E*_pp_(*t*, *τ*), as a function of gate time *t* and pump–probe delay *τ*, as well as the transmitted electric field resulting from the probe pulse, *E*_probe_(*t*), and the pump pulse, *E*_pump_(*t*, *τ*) separately, following the experimental protocols discussed above. Here, the calculated transmitted *E*-field is given by

15 $$E(t)={E}_{{\rm{THz}}}(t)-\frac{{\mu }_{0}c}{2n}J(t)\ ,$$

where *E*_THz_(*t*) is the applied THz electric field, *n* is the refractive index of the SC system, and

16 $$J=e\mathop{\sum }\limits_{{\bf{k}},\lambda }{\nabla }_{{\bf{k}}}{\xi }_{\lambda }({\bf{k}})\left[{\tilde{\rho }}_{1,1}^{(\lambda )}({\bf{k}})+{\tilde{\rho }}_{2,2}^{(\lambda )}({\bf{k}})\right]$$

is the current expressed in terms of the gauge-invariant density matrix (9). This result is obtained by solving Maxwell’s equations in a thin film geometry^38^. We then calculate the nonlinear differential transmission which is defined by Δ*E*  =  *E*_pp_(*t*, *τ*)  −  *E*_pump_(*t*, *τ*)  −  *E*_probe_(*t*) for the collinear pump–probe geometry used in the experiment. All the presented theoretical results in manuscript Fig. 4 and the supplementary are based on Δ*E* calculated as above, i.e., the signal comes from interaction of the excitations by the two phase-coherent pulses and vanishes for independent excitations. In particular, in the inset of manuscript Fig. 4a, we show Δ*E*/*E*_0_ as a function of pump–probe delay for a fixed gate time *t*, where *E*_0_ is the peak electric field strength of the applied pump E-field. The spectra of Δ*E*/*E*_0_ for different pump fluences are plotted in manuscript Fig. 4a, b, while the spectral weights of the Higgs mode resonances presented in Fig. 4c, e and the inset of Fig. 4d are extracted from Δ*E*/*E*_0_ spectra.

## Supplementary information

Supplementary Information

## Data Availability

The data that support the plots within this paper and other findings of this study are available from the corresponding author upon reasonable request.
