# Supplementary material for: Light quantum control of persisting Higgs modes in iron-based superconductors
Source: Nat Commun. 2021 Jan 11;12:258. doi: 10.1038/s41467-020-20350-6 (PMC7801641; doi:10.1038/s41467-020-20350-6)
Supplement: Supplementary file 1 — Supplementary Information [file 41467_2020_20350_MOESM1_ESM.pdf]

Supplementary Information

Light Quantum Control of Persisting Higgs Modes in Iron-Based Superconductors

C. Vaswani[1†], J. H. Kang[2†], M. Mootz[3†], L. Luo[1], X. Yang[1], C. Sundahl[2], D. Cheng[1], C. Huang[1], R. H. J. Kim[1], Z. Liu[1], Y. G. Collantes[4], E. E. Hellstrom[4], I. E. Perakis[3], C. B. Eom[2] and J. Wang[1]
[1]Department of Physics and Astronomy, Iowa State University, and Ames Laboratory, Ames, IA 50011 USA
[2]Department of Materials Science and Engineering, University of Wisconsin-Madison, Madison, WI 53706, USA
[3]Department of Physics, University of Alabama at Birmingham, Birmingham, AL 35294-1170, USA
[4]Applied Superconductivity Center, National High Magnetic Field Laboratory, Florida State University, Tallahassee, FL 32310, USA
†Equal contribution
(Dated: November 14, 2020)

Supplementary Note 1: IRON PNICTIDE EPITAXIAL THIN FILMS AND CRYSTALLINE QUALITY

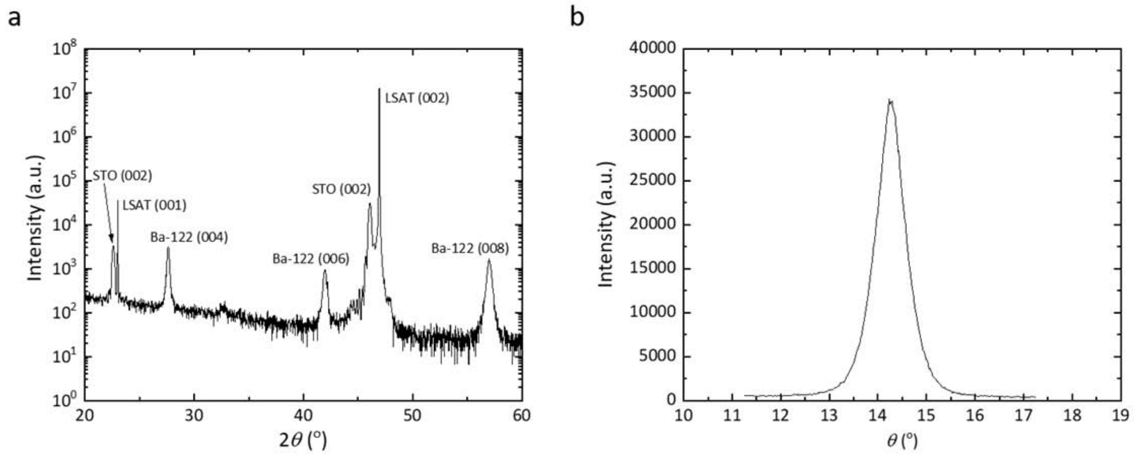

Supplementary Figure 1: (a) Out-of-plane θ-2θ XRD patterns of Co-doped $BaFe_2As_2$ films grown on $SrTiO_3$ buffered (001)-oriented (La;Sr)(Al;Ta)O_3 (LSAT) single-crystal substrates. (b) Rocking curve and FWHM for (004) reflection of Ba-122 films.

Co-doped $BaFe_2As_2$ (Ba-122) epitaxial thin films were grown on 40 nm thick $SrTiO_3$ (STO) buffered (001)-oriented (La;Sr)(Al;Ta)O_3 (LSAT) single-crystal substrates [1–3]. The LSAT substrates are double-side polished for optical measurement. The base pressure is below 3×10^{-5} Pa and the films were synthesized by pulsed laser deposition with a KrF (248 nm) ultraviolet excimer laser in a vacuum of 3×10^{-4} Pa at 730°C (growth rate: 2.4 nm/sec). The Co-doped Ba-122 target was prepared by solid-state reaction with a nominal composition of Ba/Fe/Co/As = 1:1.84:0.16:2.2. This composition was determined by wavelength-dispersive X-ray spectroscopy (WDS) measurements to produce 8% optimal doping in the grown films.

The epitaxial and crystalline quality of the optimally doped $BaFe_2As_2$ thin films was measured by four-circle X-ray diffraction (XRD). Supplementary Fig. 1(a) shows the out-of-plane θ-2θ scans of the films on STO buffered (001)-oriented LSAT single-crystal substrates. The XRD patterns show that the film 001 reflections dominate indicating c-axis growth normal to the substrate. As shown in Supplementary Fig. 1(b), the full width half maximum (FWHM) of the (004) reflection rocking curve of the films on STO/LSAT is as narrow as 0.7°, which means high quality epitaxial thin films.

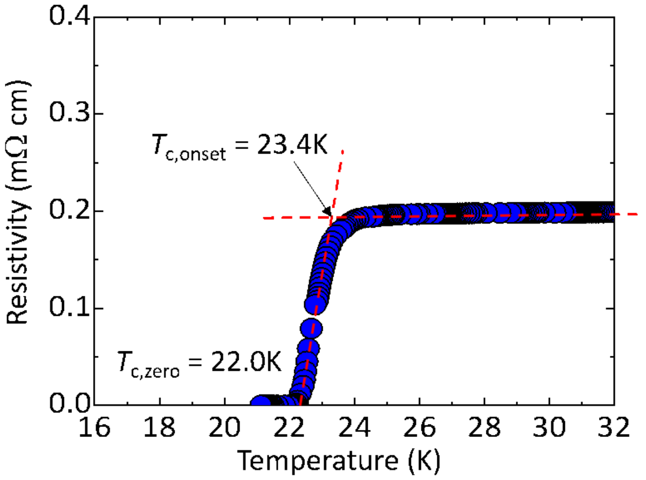

Supplementary Figure 2: Superconducting transition of Co-doped Ba-122 films; $T_{c,onset}$ and $T_{c,zero}$ of the film on STO buffered LSAT are shown.

We have also performed chemical, structural and electrical characterizations of epitaxial Co-doped $BaFe_2As_2$ (Ba-122) superconducting thin films. We determined the chemical composition of Ba122 thin films by wavelength dispersive spectroscopy (WDS) analyses. The chemical composition of the thin film is found to be $Ba(Fe_{0.92},Co_{0.08})_2As_{1.8}$, which is close to the stoichiometry of Ba122 with 8 % (atomic %) optimal Co-doping. The PLD targets were made in the same way using the same nominal composition of $Ba(Fe_{0.92},Co_{0.08})_2As_{2.2}$ as reported in our previous papers [1–3]. We measured temperature-dependent electrical resistivity for superconducting transitions by four-point method (Supplementary Fig. 2). Onset T_c and T_c at zero resistivity are as high as 23.4 K and 22.0 K, respectively, and ΔT_c is as narrow as 1.4K, which are the highest and narrowest values for Ba-122 thin films. In our prior papers, one can also check a zero-field-cooled magnetization T_c and it clearly shows a diamagnetic signal by superconducting quantum interference device (SQUID) magnetometer measurements.

Supplementary Note 2: TWO-PULSE PHASE-LOCKED COHERENT NONLINEAR SPECTROSCOPY

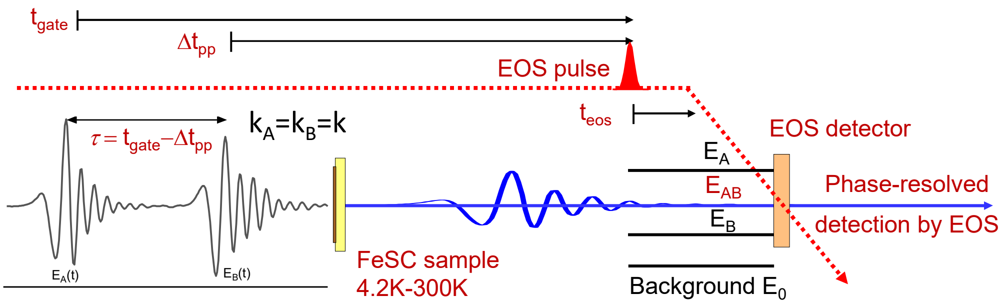

Supplementary Figure 3: A schematic of the Two-pulse phase-locked coherent nonlinear spectroscopy with phase resolved detection by electro-optic sampling in the collinear geometry[7].

Our THz pump–THz probe coherent spectroscopy experiment, illustrated in Supplementary Fig. 3, can be understood within the general framework of two-pulse phase-locked two-dimensional (2D) coherent nonlinear spectroscopy [4] and implemented with similar conditions as [5]. The experiment is performed in the collinear geometry, with two pulses E_A and E_B with wave vectors $\vec{k}_A = \vec{k}_B = \vec{k}_{\rm NL}$. The time-resolved coherent nonlinear dynamics is then explored by varying the inter-pulse delay τ between the two THz pulses. Measuring the electric fields in time-domain through electro-optic sampling (EOS) by a third pulse (red shade) allows for phase-resolved detection of the sample response. In general, such a phase-resolved two-pulse experiment can provide a number of non-linear (NL) responses as shown in the Supplementary Table 1 below [4]. The signals arise from the third order ($\chi^{(3)}$) nonlinear pump-probe responses of the superconducting state, which are separated from the linear response background. Two main contributions relevant here are measured along the same phase matching direction $\vec{k}_{\rm NL}$: (1) pump-probe (PP) signals that access condensate quench and recovery; (2) Four-wave mixing (FWM) signals that access amplitude channel coherence and/or density fluctuations. For the sake of consistency, we follow our prior publications [6] to label the time delays Δt_{pp} and t_{gate} corresponding to pulse E_A and E_B in Supplementary Fig. 3, which can be varied independently. Here, Δt_{pp} and t_{gate} are conventionally setup as $\Delta t_{pp} = t_{EOS} - t_A$ and $t_{gate} = t_{EOS} - t_B$ in order to perform the deconvolution of the polarization responses.

For the PP contribution, a polarization response $\widetilde{P}(\omega, \Delta t_{pp})$ is measured and used to obtain the time- and frequency-resolved response functions by performing deconvolution along the t_{gate} axis,

$$\widetilde{\sigma}(\omega, \Delta t_{pp}) = -i\omega\widetilde{\chi}^{(1)}(\omega, \Delta t_{pp}) = \frac{\widetilde{P}(\omega, \Delta t_{pp})}{E_B(\omega)}. \tag{1}$$

For the FWM contribution, we measure it in terms of the inter-pulse delay $\tau = t_{gate} - \Delta t_{pp}$ as

$$\Delta E_{FWM}(\tau, t_{gate}) = E_{AB}(t_g - \Delta t_{pp}, t_{gate}) - E_A(\Delta t_{pp}) - E_B(t_{gate}), \tag{2}$$

where E_{AB} means E_A and E_B are both present.

Supplementary Table 1: Contributions to $E_{NL}(\tau, t_{gate})$ and ν_0 is the carrier frequency of the THz pulses used.

		Frequency space	
NL Contributions	Wavevector space	$\nu_{t_{gate}}$	ν_τ
	$\chi^{(3)}$ effects		
Pump-probe signal	$\vec{k}_A - \vec{k}_A + \vec{k}_B = \vec{k}_{NL}$	ν_0	$-\nu_0$
	$\vec{k}_B - \vec{k}_B + \vec{k}_A = \vec{k}_{NL}$	ν_0	0
Four wave mixing	$2\vec{k}_A - \vec{k}_B = \vec{k}_{NL}$	ν_0	$+1\nu_0$
	$2\vec{k}_B - \vec{k}_A = \vec{k}_{NL}$	ν_0	$-2\nu_0$

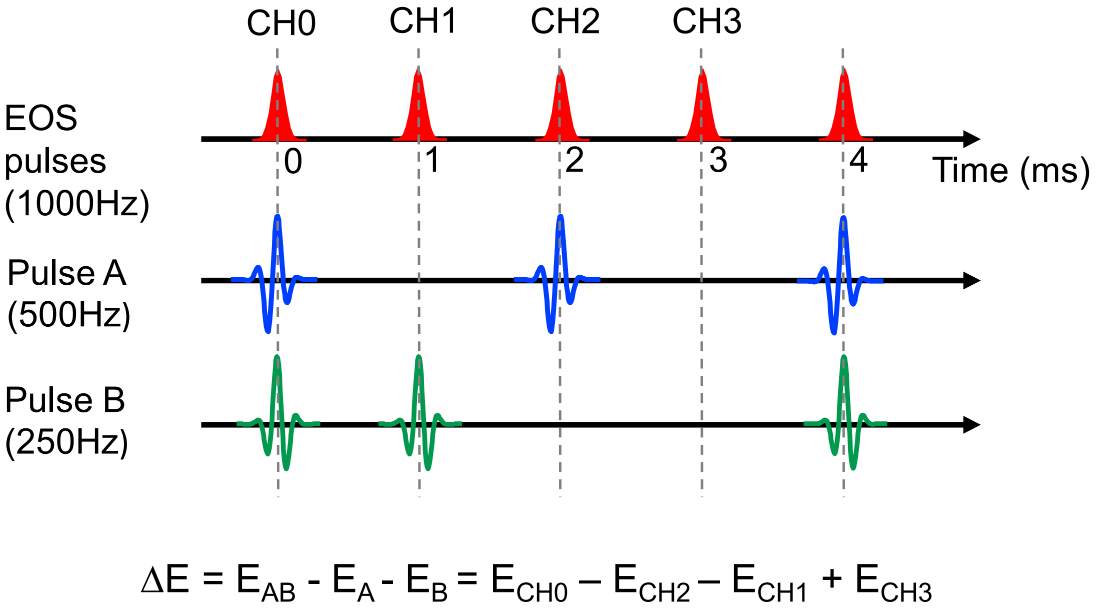

Supplementary Figure 4: A schematic of the timing sequence and data acquisition for the two-pulse phase-locked nonlinear spectroscopy experiment [6]

In our experiment, two optical choppers synchronized by the f=1kHz laser repetition rate modulate the pump and probe THz beams at f/2 (500Hz) and f/4(250Hz), respectively. The measured data is then divided into 4 channels CH0 to CH3 as shown in the schematic of the timing sequence in Supplementary Fig. 4. CH0 corresponds to the pump THz pulses as well as probe THz pulses passing through the sample that correspond to E_{AB}. Checking the pulse distribution among channels, CH1 and CH2 measure E_B and E_A. Finally, CH3 measures the background instrument noise E_0. To avoid non-linearities in the EOS detection scheme by the intense THz pulse due to over-rotation[8], we attenuate the THz pulses after the sample by multiple wire grid polarizers. After the data acquisition in each channel, we obtain different background-free nonlinear signals as

$$\Delta E = E_{AB} - E_A - E_B = E_{CH0} + E_{CH1} - E_{CH2} - E_{CH3}. \tag{3}$$

The experiment is driven by a Ti:Sapphire regenerative amplifier that delivers 3W, 35fs pulses at a 1kHz repetition rate, which are centered at 800nm. The generation of intense broadband near-single cycle pump THz pulses is achieved by tilted pulse front generation in a MgO-doped $LiNbO_3$ crystal [9, 10]. The peak pump THz field strength

generated in this manner was $\sim$ 838kV/cm for 2W of the 800nm amplifier beam, as determined by using a calibrated pyroelectric detector. The remaining portion of the amplifier beam was used for the generation of second THz pulses in a 1mm thick 110-cut ZnTe crystal. A small fraction of the laser beam was split-off for use as an optical gate pulse for electro-optic sampling in a 1mm thick 110-cut ZnTe crystal. The THz pump spot size on the sample was $\sim$1mm. The THz field strength was controlled by putting 3 wire grid polarizers in the pump path. All experiments were carried out in a continuous flow optical cryostat. The setup was continuously purged with dry air to eliminate the absorption of THz radiation by water-vapor [6, 11].

Supplementary Note 3: THZ PP SIGNALS: TIME-DEPENDENT COMPLEX CONDUCTIVITY SPECTRA

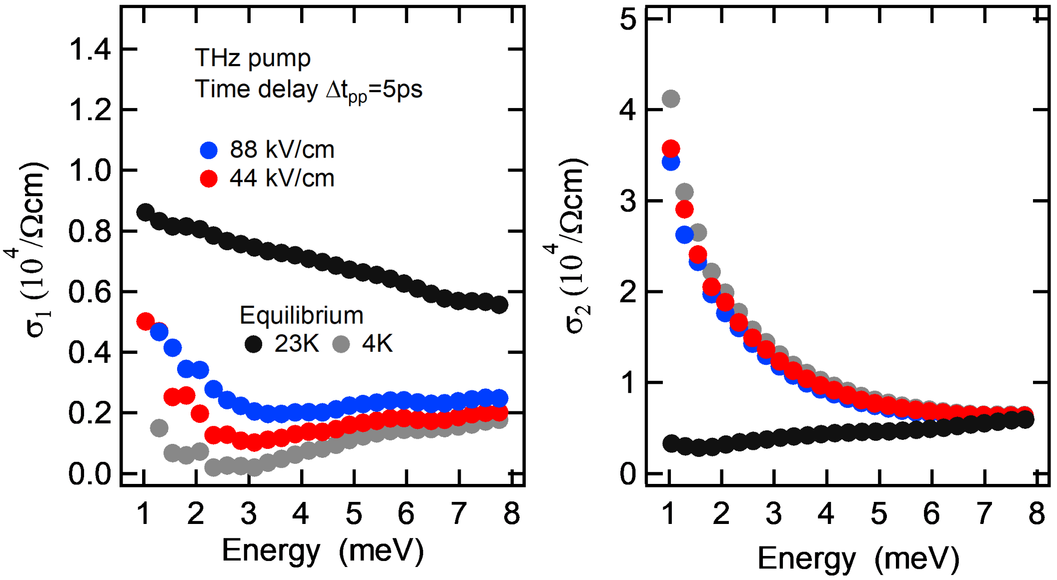

Supplementary Figure 5: Complex conductivity spectra under equilibrium and non-equilibrium conditions. The normal state(23K) and superconducting state(4.2K) spectra in equilibrium are shown in black and gray respectively. The blue and red traces indicate the spectra 5ps after the THz pump(at 4.2K) for field strengths of 88kV/cm and 44kV/cm respectively.

Unlike for the Fourier spectra of coherent FWM signals of main interest here, PP signals (Table I and Equation #1, supplementary section S2) directly access the time-dependent complex conductivity spectra, i.e., real and imaginary parts, $\sigma_1(\omega, \Delta t_{PP})$, $\sigma_2(\omega, \Delta t_{PP})$, shown in Supplementary Fig. 5 for a fixed time delay $\Delta t_{PP} = 5ps$. PP signal measurements extend the conventional, equilibrium complex conductivity $\sigma_1(\omega)$ and $\sigma_2(\omega)$ measurement (black and gray circles, Supplementary Fig. 5) to characterize the non-equilibrium post-THz-quench superconducting states. $\sigma_1(\omega, \Delta t_{PP})$, $\sigma_2(\omega, \Delta t_{PP})$ are shown for E-field = 44 kV/cm (red circles) and E-field = 88 kV/cm (blue circles) in Supplementary Fig. 5. The static conductivity can be fitted with Drude-like behavior of quasi-particles in the normal state (black circles) and condensate gaps of $2\Delta_1 = 6.8meV$ in the SC state (red circles) (discussed later in Supplementary Fig. 7). The PP conductivity spectra identify the minimal quenching of condensate density of few % for 10s of kV/cm E field used in observing coherent oscillations. Time-dependence of superfluid density can be directly obtained by the diverging $\sigma_2(\omega, \Delta t_{PP}) \propto n_s/\omega$ in Supplementary Fig. 5.

We plot Supplementary Fig. 6(a) from three representative line-cuts in Fig. 3a (main text) and then compare this with the condensate fraction (Supplementary Fig. 6(b)) extracted from the usual diverging response in $\sigma_2(\omega)$ (Supplementary Fig. 6(c)). It is clear that, after the oscillation, $\Delta E/E_0$ closely follows the pump-induced change in condensate density, $\Delta n_s/n_0$. For example, for weak THz excitation E_{pump} =56 kV/cm and below T_c, the THz pump pulse only reduces n_s slightly, e.g., $\Delta E/E_0 \propto \Delta n_s/n_0 \sim -3$ %.

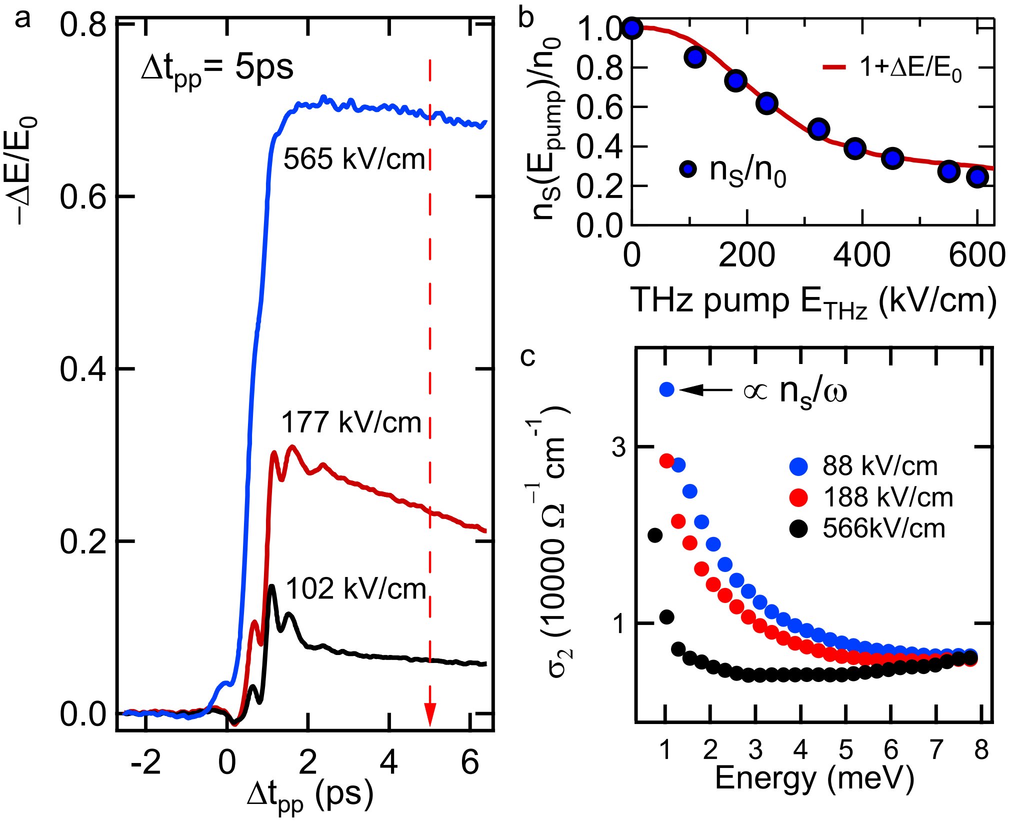

Supplementary Figure 6: (a) Differential transmission ΔE/E for three representative field strengths of 102 kV/cm(black), 177kV/cm(red) and 565kV/cm(blue). (b) Shows the condensate fraction(blue circles) $n_s(E_{pump})/n_0$ as a function of THz pump field strength, obtained from fits to $\sigma_2(\omega)$. Comparing the condensate fraction with 1+ΔE/E(red), it is evident that –ΔE/E corresponds to the fractional change in superfluid density $\Delta n_s/n_0$. (c) Illustrates $\sigma_2(\omega)$ after the pump, for THz pump field strengths of 88kV/cm(blue), 188kV/cm(red) and 566kV/cm(black).

Supplementary Note 4: THZ FWM SIGNALS: LIGHT-DRIVEN COHERENT OSCILLATIONS

Coherent oscillations with frequency ∼6.9 meV from the Fourier spectrum of the coherent time-dependent FWM signals is in excellent agreement with the lowest SC gap values $2\Delta_1 \sim 6.8meV$ obtained in our THz conductivity data using a Mattis-Bardeen approach similar to that used in [12, 13]. Specifically, Supplementary Fig. 7 plots together the conductivity of our single-crystal film sample and prior FTIR conductivity of single crystal samples at similar doping [13]. Their excellent agreement attests to the high quality of the crystalline films used and shows similar superconducting energy gaps of both samples. For example, the two superconducting energy gaps obtained from the FTIR data are $2\Delta_1 \sim 6.2meV$, that agrees with our $2\Delta_1$ value, and $2\Delta_2 \sim 14.8meV$.

The detection bandwidth for both FWM signals and PP signals are not only determined by the THz pulse durations, but are also affected by the substrate (LSAT) absorption of our thin film samples. The latter has an even bigger influence for the transient conductivity spectra due to technical details, since a comparison is needed between the transmitted E fields between the samples and substrate. There shows very large noise in the time-dependent complex conductivity $\sigma_1(\omega, \Delta t_{PP})$, $\sigma_2(\omega, \Delta t_{PP})$ above 10 meV, due to very poor signal to noise ratio there due to the LSAT substrate absorption loss. In comparison, the coherent temporal detection in Fig. 1 (main text) has slightly higher spectral range due to the direct time domain detection scheme, ∼11-12 meV.

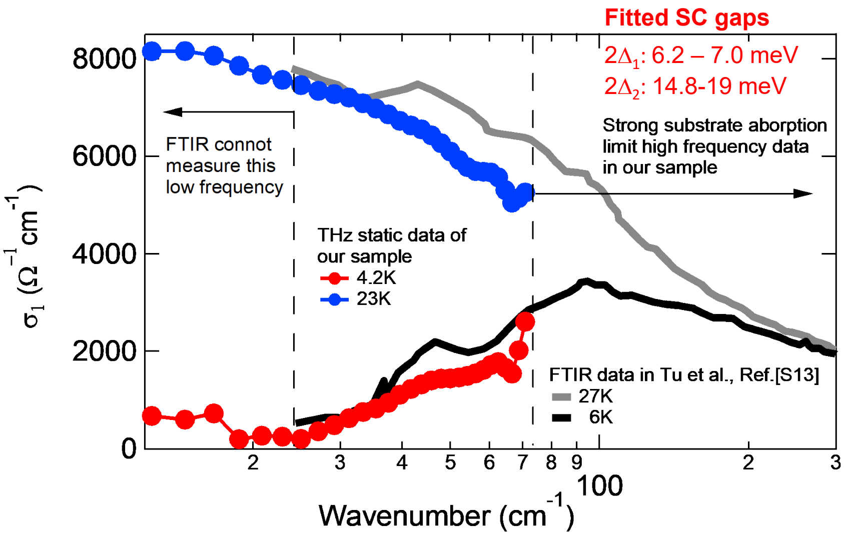

Supplementary Figure 7: A comparison between THz conductivity data of our sample (circles) at 4.2K and 23K and prior FTIR conductivity of single crystal samples (solid lines) [13].

Supplementary Note 5: OPTICAL AND ELECTRONIC PROPERTIES OF Co-DOPED $BaFe_2As_2$

It has already been established that up to five superconducting energy gaps can exist in FeSCs and can be organized into two energy scales, i.e., $2\Delta_1 \sim 7meV$ and $2\Delta_2 \sim 15meV$ [13]. Although different gaps within each of these two groups have similar magnitudes, they can still broaden the conductivity in $\sigma_1(\omega)$ around the Δ_1 gap value, due to the fact that $\sigma_1(\omega)$ becomes zero only below the smallest SC gap at sufficiently low temperature. Although the suppression of spectral weight below the superconducting critical temperature has been seen in published data [14], the Δ_1 gap is hardly seen even in these high quality data, due in part to signal to noise ratio, sample inhomogeneity, etc.

The comparison of our data with the literature shows the very high quality of both our characterization techniques in comparison with the literature and quality of our thin film samples used. Our equilibrium data is fully consistent with the reported properties of FeSCs in the literature. An example is shown in Supplementary Fig. 7. The analysis of THz conductivity spectra of our optimally doped Ba122 thin film allows the determination of key equilibrium electrodynamics and transport parameters. First, we can obtain the plasma frequency ω_p, obtained by fitting the normal state conductivity spectra with the Drude model, which gives the plasma frequency to be ~194THz (~804 meV) and the scattering rate ~2.5THz (~10 meV). These measurements are in excellent agreement with bulk single crystal samples at optimal doping: a normal state Drude plasma frequency of 972 meV, scattering rate 15 meV was obtained there. Second, some other equilibrium thermodynamic quantities can also be obtained, e.g., the London penetration depth $\lambda_L = 378\ nm$, corresponding to a condensed fraction of ~50%. These are again consistent with the prior measurements of high quality bulk samples, which show ~50% of the free carriers participating in superfluidity below T_c and a penetration depth of 300nm. Third, our sample exhibits a relatively long mean field path, comparable to the coherence length, $l/\xi = \pi\tau\Delta_{SC}/\hbar \sim 1$, which indicates that our sample lies in the intermediate regime between the clean and dirty limits.

A key electronic properties of FeSCs is strong Coulomb coupling between electron and hope pockets that is well established in iron-based superconductors [14]:

- The theoretical predictions, if we assume larger intraband than interband interaction, provide the WRONG pairing symmetry, i.e., s++ instead of s+−, and the electron-phonon interaction is too small to account for the high critical temperatures demonstrated. One of the first phase-sensitive tests of the symmetry of the superconducting order parameter have revealed a sign change, which indicates the dominance of U over V. In addition, such a s+− pairing is directly corroborated by the observation of a resonance peak in the neutron scattering spectra at the SDW wave vector connecting the interband portions of the Fermi surface with the

opposite signs of the superconducting order parameter. This SDW resonant peak has indeed been seen in almost all known FeSCs, which clearly show the dominance of the interband U.

- Opposite band shifts for hole- and electron-like pockets as a function of doping are a direct consequence of the multiband character of FeSCs with a dominant interband interaction U.
- The pairing mechanism in FeSCs is believed to be SDW fluctuations which relate closely to the antiferromagnetic/nesting wave vector between e- and h-bands, i.e., interband instead of intraband, at the Fermi surface.
- The dominance of interband coupling U between e-h pockets over intraband is widely accepted by now also due to some other complementary ultrafast measurements, e.g., spin-exciton modes in ultrafast THz conductivity [15]. These have been the foundational facts, being tested extensively in the community in the past 10 years, which lead to the conclusion of the unconventional nature of the pairing due to a dominant interband interaction in Fe-based superconductivity.

Supplementary Note 6: GAUGE-INVARIANT THEORY OF THZ-LIGHT DRIVEN MULTI-DIMENSIONAL THZ COHERENT NONLINEAR SPECTROSCOPY AND NON-EQUILIBRIUM DYNAMICS OF IRON PNICTIDE SUPERCONDUCTORS

In this supplement we briefly summarize our gauge-invariant non-equilibrium density-matrix approach [11, 16, 17] used to model the THz multi-dimensional coherent nonlinear spectra of iron pnictide superconductors. We use our quantum kinetic modeling to directly simulate experimental measured quantities, i. e. the coherent FWM and PP signals calculated analogous to similar two-pulse THz signals in semiconductors [7], and not only the order parameters dynamics. The results presented here are part of the full 2D THz phase-coherent nonlinear spectroscopy calculation, where PP, FWM, HHG, and higher nonlinearities show up as distinct peaks that differ from the spectra of the order parameters, as they arise from two phase-coherent THz pulse excitations. In particular, the pump–probe and FWM responses presented in the main text crucially depend on the properties and nonlinear interferences of the two applied electric fields and corresponding excitations, i.e. they depend on fluence, duration, time delay, and frequency of the applied pump and probe phase-coherent pulses. The above also depends on the bandstructure, in particular on the multi-pocket structure of FeSCs where strong interband coupling dominates over intra-pocket interaction as discussed below.

We start from the microscopic spatial-dependent Boguliobov–de Gennes Hamiltonian for multi-band superconductors [17–19]

$$H = \sum_{\nu,\alpha} \int \mathrm{d}^3\mathbf{x}\, \psi^\dagger_{\alpha,\nu}(\mathbf{x}) \left[\xi_\nu(\mathbf{p} + e\mathbf{A}(\mathbf{x},t)) - \mu - e\phi(\mathbf{x},t) + \mu_{\mathrm{F}}^{\alpha,\nu}(\mathbf{x})\right] \psi_{\alpha,\nu}(\mathbf{x}) \\ - \sum_{\nu} \int \mathrm{d}^3\mathbf{x} \left[\Delta_\nu(\mathbf{x}) \psi^\dagger_{\uparrow,\nu}(\mathbf{x}) \psi^\dagger_{\downarrow,\nu}(\mathbf{x}) + \mathrm{h.c.}\right] . \tag{4}$$

Here the Fermionic field operators $\psi^\dagger_{\alpha,\nu}(\mathbf{x})$ and $\psi_{\alpha,\nu}(\mathbf{x})$ create and annihilate an electron with spin α in pocket ν; $\xi_\nu(\mathbf{p} + e\mathbf{A}(\mathbf{x},t))$ corresponds to the dispersion of the pocket with momentum operator $\mathbf{p} = -\mathrm{i}\nabla_{\mathbf{x}}$ ($\hbar$=1), vector potential $\mathbf{A}(\mathbf{x},t)$, and electron charge $-e$; μ is the chemical potential while $\phi(\mathbf{x},t)$ denotes the scalar potential.

The SC complex order parameter components arising from the condensates in the different Fermi sea pockets are

$$\Delta_\nu(\mathbf{x}) = -2 \sum_\lambda g_{\nu,\lambda} \langle \psi_{\downarrow,\lambda}(\mathbf{x}) \psi_{\uparrow,\lambda}(\mathbf{x}) \rangle = |\Delta_\nu(\mathbf{x})| \mathrm{e}^{\mathrm{i}\theta_\nu(\mathbf{x})} , \tag{5}$$

while the Fock energy is given by

$$\mu_{\mathrm{F}}^{\alpha,\nu}(\mathbf{x}) = -g_{\nu,\nu} n_{\alpha,\nu}(\mathbf{x}) , \quad n_{\alpha,\nu}(\mathbf{x}) = \langle \psi^\dagger_{\alpha,\nu}(\mathbf{x}) \psi_{\alpha,\nu}(\mathbf{x}) \rangle , \tag{6}$$

and ensures charge conservation in the SC system. Here $g_{\lambda,\nu}$ is the effective inter ($\lambda \neq \nu$) and intra ($\lambda = \nu$) electron–electron interaction in the BCS theory developed in previous works.

Hamiltonian (4) is gauge-invariant under the gauge transformation [20]

$$\Psi_\nu(\mathbf{x}) \rightarrow \mathrm{e}^{\mathrm{i}\tau_3 \Lambda(\mathbf{x})/2} \Psi_\nu(\mathbf{x}) \tag{7}$$

when vector potential, scalar potential, and different order parameter component phases transform as

$$\mathbf{A}(\mathbf{x}) \rightarrow \mathbf{A}(\mathbf{x}) + \frac{1}{2e}\nabla\Lambda(\mathbf{x}), \quad \phi(\mathbf{x}) \rightarrow \phi(\mathbf{x}) - \frac{1}{2e}\frac{\partial}{\partial t}\Lambda(\mathbf{x}), \quad \theta_\nu(\mathbf{x}) \rightarrow \theta_\nu(\mathbf{x}) + \Lambda(\mathbf{x}), \tag{8}$$

with the field operator for band ν in Nambu space $\Psi_\nu(\mathbf{x}) = (\psi_{\uparrow,\nu}(\mathbf{x}), \psi^\dagger_{\downarrow,\nu}(\mathbf{x}))^T$ and Pauli spin matrix $\tau_3 = \begin{pmatrix} 1 & 0 \\ 0 & -1 \end{pmatrix}$. The conventional density matrix describing band ν, $\rho^{(\nu)}(\mathbf{x}, \mathbf{x}') = \langle \Psi_\nu(\mathbf{x})^\dagger \Psi_\nu(\mathbf{x}')\rangle$, depends on the choice of the gauge. To simplify the gauge transformation of the density matrix, we define center-of-mass and relative coordinates $\mathbf{R} = (\mathbf{x} + \mathbf{x}')/2$ and $\mathbf{r} = \mathbf{x} - \mathbf{x}'$ and introduce a new density matrix [11, 16, 17],

$$\tilde{\rho}^{(\nu)}(\mathbf{r}, \mathbf{R}) = \exp\left[-\mathrm{i}e\int_0^{\frac{1}{2}} \mathrm{d}\lambda\, \mathbf{A}(\mathbf{R} + \lambda\,\mathbf{r}, t)\cdot \mathbf{r}\,\tau_3\right] \rho^{(\nu)}(\mathbf{r}, \mathbf{R}) \exp\left[-\mathrm{i}e\int_{-\frac{1}{2}}^{0} \mathrm{d}\lambda\, \mathbf{A}(\mathbf{R} + \lambda\,\mathbf{r}, t)\cdot \mathbf{r}\,\tau_3\right], \tag{9}$$

where $\rho^{(\nu)}(\mathbf{r}, \mathbf{R}) = \langle \Psi^\dagger_\nu(\mathbf{R} + \frac{\mathbf{r}}{2})\Psi_\nu(\mathbf{R} - \frac{\mathbf{r}}{2}))\rangle$. This new density matrix $\tilde{\rho}^{(\nu)}(\mathbf{r}, \mathbf{R})$ transforms as [11, 16]

$$\tilde{\rho}^{(\nu)}(\mathbf{r}, \mathbf{R}) \rightarrow \exp\left[\mathrm{i}\tau_3\Lambda(\mathbf{R})/2\right] \tilde{\rho}^{(\nu)}(\mathbf{r}, \mathbf{R}) \exp\left[-\mathrm{i}\tau_3\Lambda(\mathbf{R})/2\right] \tag{10}$$

under the gauge transformation (7), where the transformed phase $\Lambda(\mathbf{R})$ only depends on the center-of-mass coordinate and not on both coordinates $\mathbf{R}$ and $\mathbf{r}$ as in the original density matrix. This property simplifies the gauge-invariant description of the photo-excited non-equilibrium SC dynamics.

The equation of motion for $\tilde{\rho}^{(\nu)}(\mathbf{r}, \mathbf{R})$ is now derived by using the Heisenberg equation of motion technique. To simplify the equations of motion, we Fourier transform the obtained exact results with respect to the relative coordinate $\mathbf{r}$ and then apply a gradient expansion which is valid for SC systems with condensate center–of–mass spatial fluctuations smoother than the spatial dependence of Cooper pair relative motion. To simplify the problem further, we eliminate the phase of the order parameter $\Delta_{\nu_0}(\mathbf{R})$ by applying the gauge transformation [11, 16, 17]

$$\tilde{\rho}^{(\nu)}(\mathbf{k}, \mathbf{R}) = \mathrm{e}^{-\mathrm{i}\tau_3\theta_{\nu_0}(\mathbf{R})/2}\tilde{\rho}^{(\nu)}(\mathbf{k}, \mathbf{R})\mathrm{e}^{\mathrm{i}\tau_3\theta_{\nu_0}(\mathbf{R})/2}. \tag{11}$$

After assuming a homogeneous SC system and homogeneous excitation conditions by neglecting $\mathbf{R}$-dependence, we obtain the gauge-invariant Bloch equations for multi-band superconductors that were solved numerically here [11, 16, 17]

$$\begin{aligned}
\mathrm{i}\frac{\partial}{\partial t}\tilde{\rho}^{(\nu)}_{1,1}(\mathbf{k}) &= -\mathrm{i}\,e\,\mathbf{E}(t)\cdot\nabla_{\mathbf{k}}\tilde{\rho}^{(\nu)}_{1,1}(\mathbf{k}) - |\Delta_\nu|\left[\mathrm{e}^{\mathrm{i}\,\delta\theta_\nu}\tilde{\rho}^{(\nu)}_{1,2}(\mathbf{k} - \mathbf{p}_\mathrm{S}/2) - \mathrm{e}^{-\mathrm{i}\,\delta\theta_\nu}\tilde{\rho}^{(\nu)}_{2,1}(\mathbf{k} - \mathbf{p}_\mathrm{S}/2)\right], \\
\mathrm{i}\frac{\partial}{\partial t}\tilde{\rho}^{(\nu)}_{2,2}(\mathbf{k}) &= \mathrm{i}\,e\,\mathbf{E}(t)\cdot\nabla_{\mathbf{k}}\tilde{\rho}^{(\nu)}_{2,2}(\mathbf{k}) + |\Delta_\nu|\left[\mathrm{e}^{\mathrm{i}\,\delta\theta_\nu}\tilde{\rho}^{(\nu)}_{1,2}(\mathbf{k} + \mathbf{p}_\mathrm{S}/2) - \mathrm{e}^{-\mathrm{i}\,\delta\theta_\nu}\tilde{\rho}_{2,1}(\mathbf{k} + \mathbf{p}_\mathrm{S}/2)\right], \\
\mathrm{i}\frac{\partial}{\partial t}\tilde{\rho}^{(\nu)}_{1,2}(\mathbf{k}) &= -[\xi_\nu(\mathbf{k} - \mathbf{p}_\mathrm{S}/2) + \xi_\nu(-\mathbf{k} - \mathbf{p}_\mathrm{S}/2) + 2(\mu_\mathrm{eff} + \mu^\nu_\mathrm{F})]\tilde{\rho}^{(\nu)}_{1,2}(\mathbf{k}) \\
&\quad + |\Delta_\nu|\mathrm{e}^{-\mathrm{i}\,\delta\theta_\nu}\left[\tilde{\rho}^{(\nu)}_{2,2}(\mathbf{k} - \mathbf{p}_\mathrm{S}/2) - \tilde{\rho}^{(\nu)}_{1,1}(\mathbf{k} + \mathbf{p}_\mathrm{S}/2)\right].
\end{aligned} \tag{12}$$

Here we introduced the gauge-invariant superfluid momentum

$$\mathbf{p}_\mathrm{S} = -2\,e\,\mathbf{A} \tag{13}$$

and effective chemical potential

$$\mu_\mathrm{eff} = e\,\phi + \frac{1}{2}\frac{\partial}{\partial t}\theta_{\nu_0} - \mu. \tag{14}$$

The Leggett mode corresponds to oscillations of the phase difference

$$\delta\theta_\nu = \theta_{\nu_0} - \theta_\nu, \tag{15}$$

while the Higgs mode is defined by the amplitude oscillation of the multi-component complex SC order parameter. The latter is expressed in terms of the gauge invariant density matrix (9). To lowest order in the gradient expansion [11, 16],

$$|\Delta_\nu| = -2\mathrm{e}^{-\mathrm{i}\,\delta\theta_\nu}\sum_\lambda g_{\nu,\lambda}\tilde{\rho}^\lambda_{2,1}(\mathbf{k}). \tag{16}$$

There are three mechanisms contributing to the driving of the Higgs modes [17]. First, quantum transport contributions $\propto \mathbf{E}$ in the equations of motion (12) lead to an acceleration of the Cooper-pair condensate by the pump electric field(Lightwave Quantum Electronics)

$$\frac{\partial}{\partial t}\mathbf{p}_{\mathrm{S}} = 2e\,\mathbf{E}\,, \tag{17}$$

which is neglected in the Anderson pseudo-spin model. This results in SC order parameter nonlinearities that are of odd order in the electric field. The condensate acceleration breaks equilibrium-inversion symmetry of the SC system and can lead to dc supercurrent generation when lightwave propagation effects are included [17]. Second, the minimal coupling, $\xi(\mathbf{k}-\mathbf{p}_{\mathrm{S}}/2)+\xi(\mathbf{k}+\mathbf{p}_{\mathrm{S}}/2)$, known from the Anderson pseudo-spin model drives even-order nonlinearities of the SC order parameter and depends on the band dispersion non-parabolicity. Third, the induced condensate momentum leads to a displacement of populations and coherences in momentum space by $\mathbf{p}_{\mathrm{S}}/2$ in the equations of motion which is also neglected in the Anderson pseudospin model. While the linear coupling to the superconductor via the quantum transport terms dominate the driving of the Higgs modes in the perturbative excitation regime [19], the excitation of the hybrid Higgs mode in the non-perturbative regime is dominated by the quadratic $\mathbf{p}_{\mathrm{S}}^2$-coupling known from the Anderson pseudo-spin model [17].

In our calculations we solve the gauge-invariant optical Bloch equations (12) for a 3-pocket model with a hole (h) pocket centered at the Γ-point and two electron (e) pockets located at $(\pi,0)$ and $(0,\pi)$. We include the inter e-h pocket interactions ($U = g_{\mathrm{e,h}} = g_{\mathrm{h,e}}$) as well as intra-pocket interactions ($V_\lambda = g_{\lambda,\lambda}$) while inter e-e pocket interactions are neglected for simplicity. The dominance of interband coupling U between e-h pockets over intraband in Fe-based SCs is taken into account by using an interband-to-intraband interaction ratio of $r = U/V = 10$. The pockets are modeled using the square lattice nearest-neighbor tight-binding dispersion $\xi_\nu(\mathbf{k}) = -2\,[J_{\nu,x}\cos(k_x)+J_{\nu,y}\cos(k_y)]+\mu_\nu$ with hopping parameter $J_{\nu,i}$ and band-offset μ_ν. We choose a circular hole pocket with $J_{1,x} = J_{1,y} = 10.0$ meV and $\mu_1 = -37.5$ meV. We introduce the known particle-hole asymmetry between electron and hole pockets in our system [21, 22, 24] by considering elliptical electron pockets with $J_{2,x} = J_{3,y} = -10.0$ meV, $J_{2,y} = J_{3,x} = -50.0$ meV, and $\mu_2 = \mu_3 = 57.5$ meV. The latter can lead to coexistence of superconductivity and spin-density wave by changing the doping level [25]. For the doping levels considered here, such asymmetry in our calculation strongly suppresses the $\omega_{\mathrm{H},2}$ mode in the spectra of coherent $\Delta E/E$ dynamics presented in Figs. 4(a)-(b) in the main text as discussed in more detail below. We assume $s\pm$-pairing symmetry with equilibrium SC order parameters $\Delta_1 = 3.4\,\mathrm{meV}$ for the hole pocket and $\Delta_2 = \Delta_3 = 9.7\,\mathrm{meV}$ for the electron pockets. To directly model our phase-coherent nonlinear pump–probe spectroscopy experiments analogous to 2D THz phase-coherent nonlinear spectroscopy in semiconductors [7], we calculate the nonlinear differential transmission, ΔE, and not just the order-parameter dynamics. The nonlinear differential transmission $\Delta E/E_0$ is obtained by computing the transmitted E-field of both pump and probe pulse, $E_{\mathrm{pp}}(t,\tau)$, as a function of gate time t and pump–probe delay τ, as well as the transmitted electric field resulting from the probe pulse, $E_{\mathrm{probe}}(t)$, and the pump pulse, $E_{\mathrm{pump}}(t,\tau)$ separately, following the experimental protocols discussed above. Here, the calculated transmitted E-field is given by

$$E(t) = E_{\mathrm{THz}}(t) - \frac{\mu_0 c}{2n} J(t)\,, \tag{18}$$

where $E_{\mathrm{THz}}(t)$ is the applied THz electric field, n is the refractive index of the SC system, and

$$J = e\sum_{\mathbf{k},\lambda} \nabla_{\mathbf{k}}\xi_\lambda(\mathbf{k})\left[\tilde{\rho}^{(\lambda)}_{1,1}(\mathbf{k}) + \tilde{\rho}^{(\lambda)}_{2,2}(\mathbf{k})\right] \tag{19}$$

is the current expressed in terms of the gauge-invariant density matrix (12). This result is obtained by solving Maxwell's equations in a thin film geometry [17]. We then calculate the nonlinear differential transmission which is defined by $\Delta E = E_{\mathrm{pp}}(t,\tau) - E_{\mathrm{pump}}(t,\tau) - E_{\mathrm{probe}}(t)$ for the collinear pump–probe geometry used in the experiment. All the presented theoretical results in manuscript Fig. 4 and the supplementary are based on ΔE calculated as above, i. e. the signal comes from interaction of the excitations by the two phase-coherent pulses and vanishes for independent excitations. In particular, in the inset of manuscript Figs. 4(a), we show $\Delta E/E_0$ as a function of pump–probe delay for a fixed gate time t, where E_0 is the peak electric field strength of the applied pump E field. The spectra of $\Delta E/E_0$ for different pump fluences are plotted in manuscript Figs. 4(a) and (b), while the spectral weights of the Higgs mode resonances presented in Figs. 4(c) and (e) and the inset of Fig. 4(d) are extracted from $\Delta E/E_0$ spectra.

We first study the order parameter dynamics in the perturbative excitation regime. Therefore, we calculate the spectral functions $H_i(\omega)$ defined in Eq. (39) of Ref. [23] which are defined by $\Delta_i'(\omega) = \frac{\mathbf{p}_{\mathrm{S}}^2}{2}H_i(\omega)$ and are obtained by linearizing the Anderson pseudo-spin equations with respect to deviations from the equilibrium state. Here Δ_i' is the

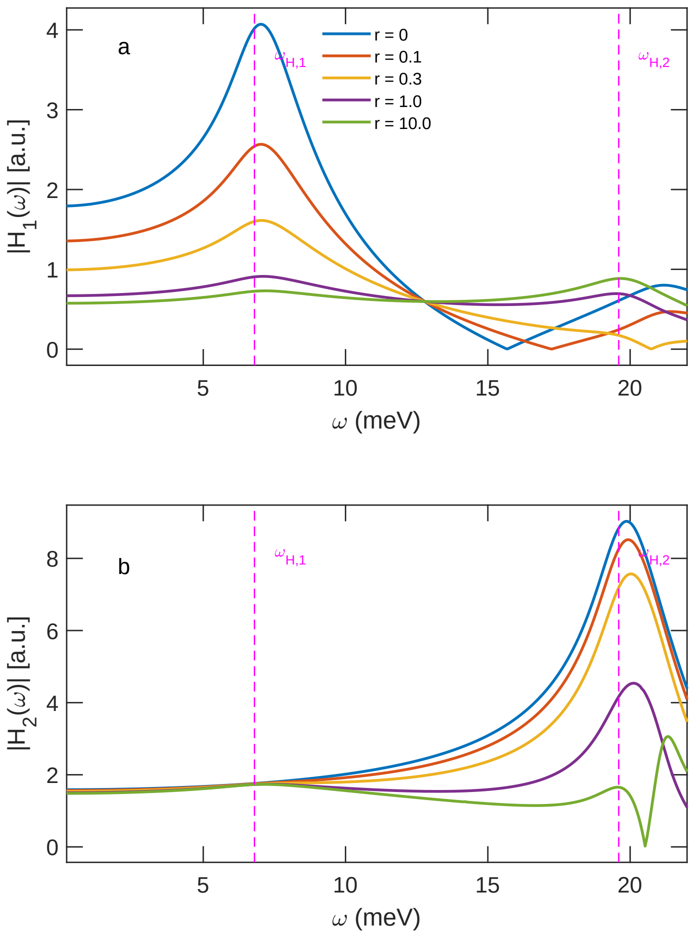

Supplementary Figure 8: Order parameter dynamics in the perturbative excitation regime. Spectral functions $|H_i(\omega)|$ defined in Eq. (39) of Ref. [23] are shown for (a) the h-pocket ($i = 1$) and (b) the e-pockets ($i = 2$) for different interband $r = U/V$. Vertical dashed lines indicate the Higgs mode energies.

real part of the complex-valued order parameter Δ_i which directly monitors the Higgs mode dynamics. To calculate $H_i(\omega)$ for different interband interactions, we self-consistently solve the SC gap equations for fixed gap values to obtain the intraband interactions V for any given $r = U/V$. By inserting the resulting V and corresponding U into Eq. (39) of Ref. [23] we can directly study the effect of the interband U on $H_i(\omega)$. The resulting spectral functions are plotted in Supplementary Fig. 8 for different interband couplings r. Without interband interaction, $H_i(\omega)$ has only a peak at $\omega_{\mathrm{H,i}}$ as expected. For finite interband U, spectral weight is transferred between the Higgs modes. As a result, a peak appears at $\omega_{\mathrm{H,2}}$ in $H_1(\omega)$ and a small peak emerges at $\omega_{\mathrm{H,1}}$ in $H_2(\omega)$ with increasing interband U. At the same time, the $\omega_{\mathrm{H,i}}$-mode in $H_i(\omega)$ spectrum decreases with increasing U such that the emerging peak exceeds the $\omega_{\mathrm{H,i}}$-mode in $H_i(\omega)$ spectrum at elevated U. The latter differs from the results presented in Ref. [23] where the peak of the $\omega_{\mathrm{H,1}}$-mode vanishes with increasing U. This is a consequence of the larger DOS of the Δ_1-pocket compared to Δ_2-pocket. The latter suppresses the $\omega_{\mathrm{H,1}}$-mode and is different to the pnictide bandstructure as discussed in more detail below. For the strong interband interaction of $r = 10$ used in the simulations in the manuscript, both Higgs modes have a comparable spectral weight in $H_i(\omega)$. Since the lower Higgs mode $\omega_{\mathrm{H,1}}$ is stronger excited by the E- as well as E^2-pump field in our study, the order parameter spectra show a pronounced peak at the lower Higgs mode and only a small peak at the higher Higgs mode in the perturbative-excitation regime as demonstrated next.

To illustrate the difference between order parameter dynamics and two-pulse coherent $\Delta E/E$ response, Supplemen-

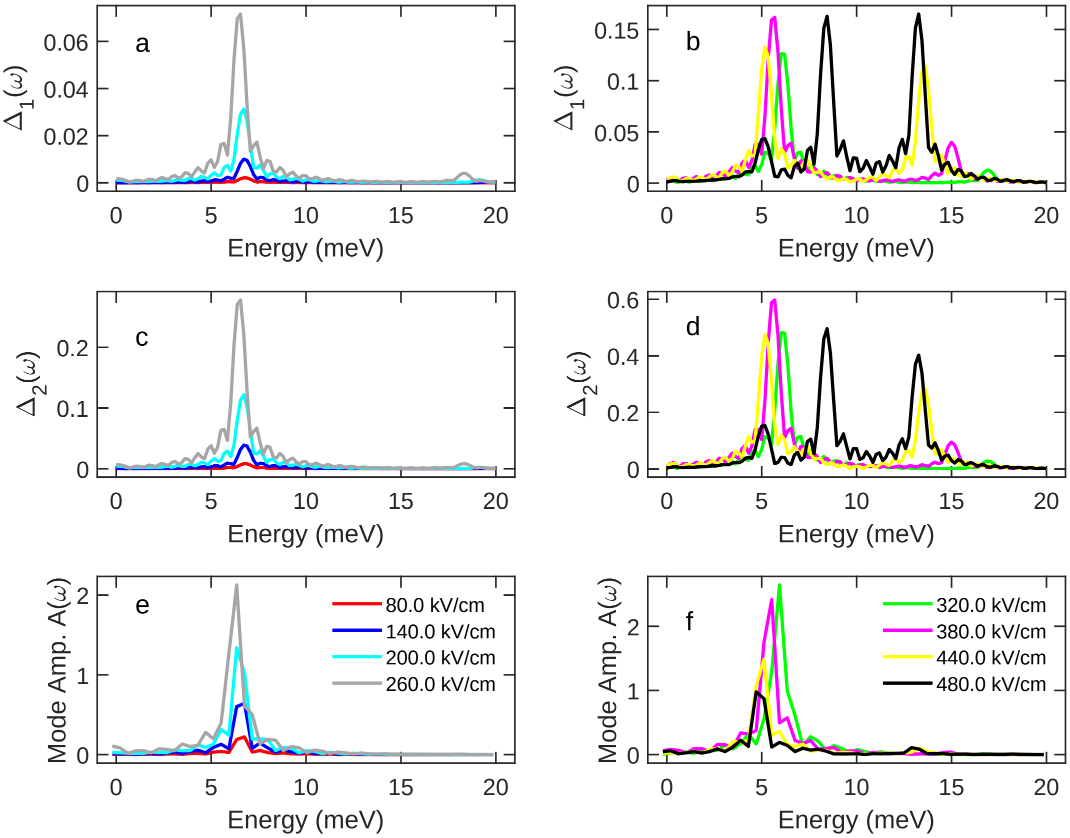

Supplementary Figure 9: Order parameter dynamics vs. coherent FWM signal. **a**, Calculated $\Delta_1(\omega)$ spectra for low pump fields show an increase in amplitude of the Higgs mode $\omega_{\mathrm{H},1}$ resonance. **b**, Calculated $\Delta_1(\omega)$ spectra for higher pump fields show a decrease and redshift of the Higgs mode $\omega_{\mathrm{H},1}$ resonance while a peak at Higgs mode $\omega_{\mathrm{H},2}$ emerges and grows with increasing pump field. At elevated pump fields an additional peak appears in between both Higgs modes at the beating energy $\omega_{\mathrm{H},2} - \omega_{\mathrm{H},1}$ which confirms the strong coupling between both Higgs modes. **c**, **d**, The corresponding $\Delta_2(\omega)$ spectra. **e**, **f**, The corresponding spectra of the coherent FWM response.

tary Fig. 9 shows the calculated $\Delta_1(\omega)$ spectra for low (Supplementary Fig. 9(a)) and high (Supplementary Fig. 9(b)) pump E-field strengths, together with the corresponding spectra of $\Delta_2(t)$ (Supplementary Figs. 9(c)-(d)) and $\Delta E/E_0$ (Supplementary Figs. 9(e)-(f)). The $\Delta_i(\omega)$ spectra show a peak at Higgs mode $\omega_{\mathrm{H},1}$ at low pump fluences, which grows up to 380.0 kV/cm. With increasing pump fluence, the amplitude of the peak at $\omega_{\mathrm{H},1}$ saturates before decreasing and red-shifting at elevated E-field strengths. In this pump field regime, a second peak emerges at Higgs mode $\omega_{\mathrm{H},2}$ due to interband coupling between the electron and hole pockets, which grows with increasing pump field strength. The strong coherent coupling between both Higgs modes is confirmed by the emergence of a peak at the beating energy $\omega_{\mathrm{H},2} - \omega_{\mathrm{H},1}$ at elevated pump fields. As a result, $\Delta_i(\omega)$ spectra also show a transfer of spectral weight between the Higgs modes, which is strongly pronounced compared to $\Delta E/E_0$ spectra.

Supplementary Note 6.1: Nonlinear Higgs spectroscopy of multi-band superconductors with vanishing interband coupling

The dominance of interband coupling U between e-h pockets over intraband in Fe-based SCs leads to spectral weight transfer between the Higgs modes which manifests itself by a decrease of the spectral weight and only a small red shift

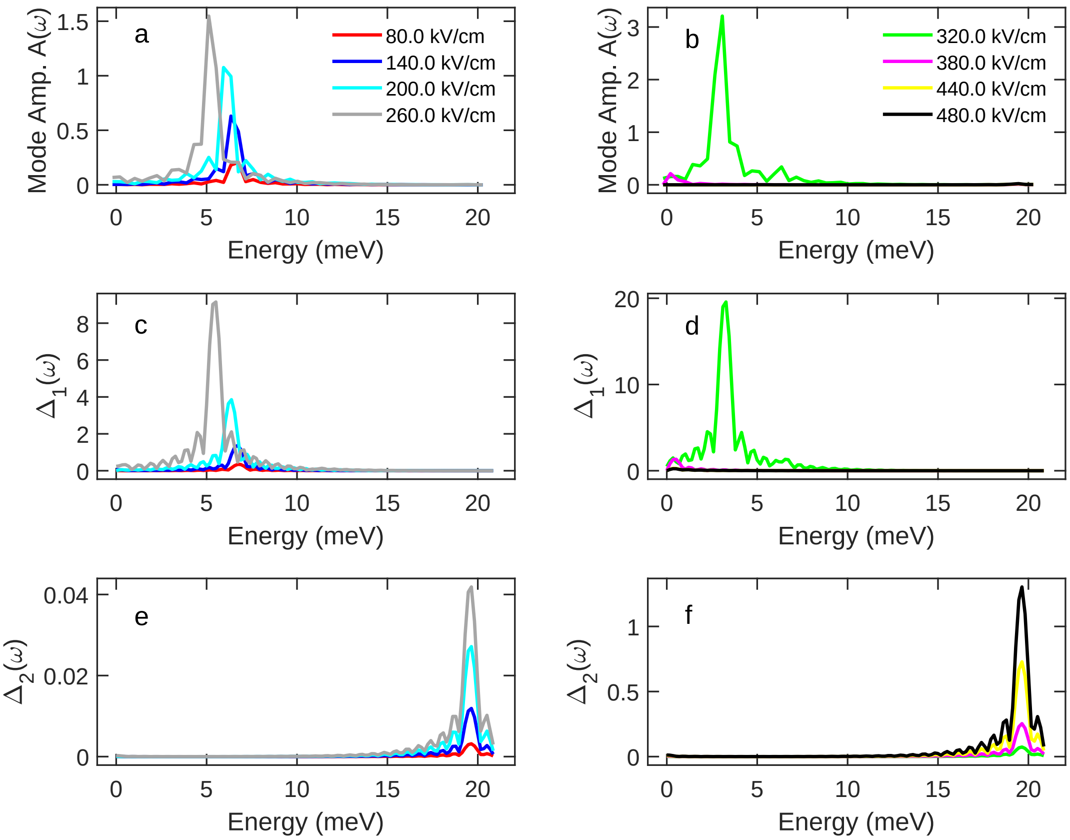

Supplementary Figure 10: Nonlinear Higgs spectroscopy of multi-band superconductor with vanishing interband interaction. **a**,**b** Calculated FWM spectra for low and high pump fields show both Higgs modes with Higgs mode $\omega_{\mathrm{H},1}$ dominating the nonlinear response. The amplitude of both modes grows monotonically with increasing pump field up to the complete quench of order parameter Δ_1 where the spectral weight of Higgs mode $\omega_{\mathrm{H},1}$ decays to zero while the one of Higgs mode $\omega_{\mathrm{H},2}$ continues growing. The corresponding calculated $\Delta_i(\omega)$ spectra for $i = 1$ (**c**,**d**) and $i = 2$ (**e**,**f**) have only a peak at $\omega_{\mathrm{H,i}}$ which grows with increasing pump field.

of Higgs mode $\omega_{\mathrm{H},1}$ at elevated pump fields, while at the same time a peak at Higgs mode $\omega_{\mathrm{H},2}$ emerges and its spectral weight grows with increasing pump fluence. The observed transfer of spectral weight between the Higgs modes is in general more pronounced and appears at much lower pump fields with increasing interband-to-intraband interaction ratio. To demonstrate that such a transfer of spectral weight is not observable without interband interaction, i.e. for $U = 0$, Supplementary Figs. 10(a) and (b) show $\Delta E/E$ spectra for low (regime I) and high pump E-field strengths (regime II) resulting from a calculation without interband coupling U. In this case, both modes are affected by the THz field, although in a different way due to spectral overlap with the pulse. In particular, the excitation of a two-band superconductor without interband interaction is comparable to the excitation of two independent one-band superconductors with different resonance conditions. Since the the broader pump E^2 spectrum used covers mainly the Higgs mode $\omega_{\mathrm{H},1}$, the FWM spectra show one pronounced peak at Higgs mode $\omega_{\mathrm{H},1}$ and only a small peak at Higgs mode $\omega_{\mathrm{H},2}$ (not observable on the shown scale) at low pump fluences. With increasing pump field Δ_1 becomes completely quenched and the spectral weight of Higgs mode $\omega_{\mathrm{H},1}$ vanishes while the peak at Higgs mode $\omega_{\mathrm{H},2}$ further grows with increasing pump field. In strong contrast to the result with finite interband interaction U, the spectral weight of both modes increases monotonically with increasing pump field up to the complete quench of Δ_1 without transferring spectral weight between the modes due to uncoupled bands. This is essentially similar to quantum quenching of the single-band BCS pairing interaction [26] that induce Higgs oscillations with amplitude

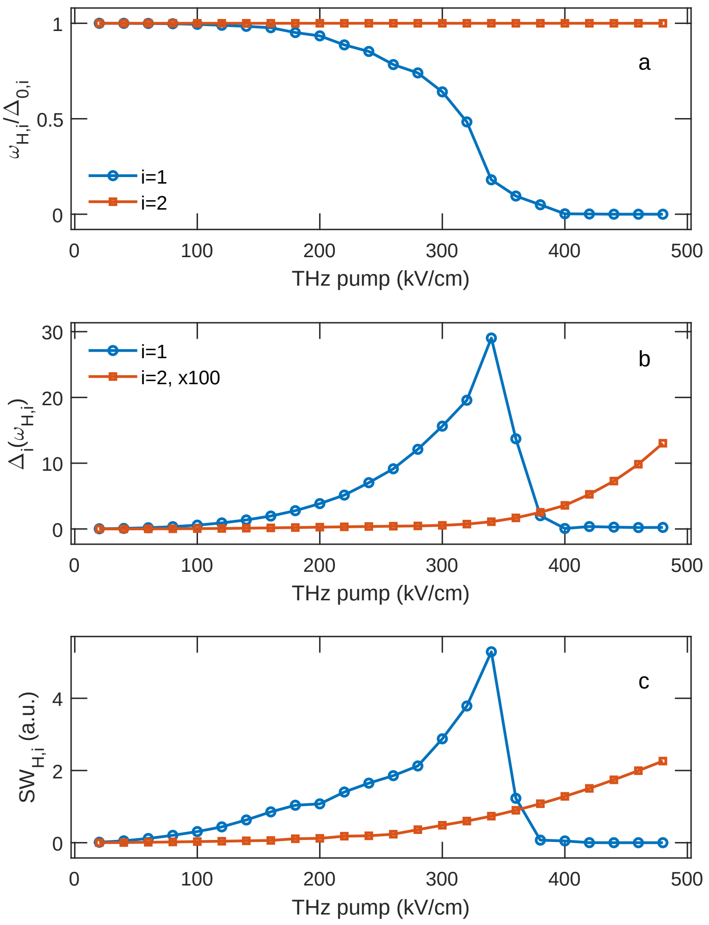

Supplementary Figure 11: Pump field dependence of Higgs mode signals for vanishing interband interaction. **a**, Pump field dependence of $\omega_{\mathrm{H,i}}$ for $i = 1$ (blue line) and $i = 2$ (red line) for $U = 0$. The corresponding pump field dependences of (**b**) $\omega_{\mathrm{H,i}}$ peak amplitude in $\Delta_i(\omega)$ spectra and (**c**) spectral weight of $\omega_{\mathrm{H,i}}$ peak in FWM spectra show a monotonic increase up to a Δ_1 quench of roughly 90%. A further increase of the pump field leads to the complete quench of the order parameter Δ_1 and a decrease of the spectral weight of Higgs mode $\omega_{\mathrm{H,1}}$ to zero while $\mathrm{SW}_{\mathrm{H,2}}$ is further increasing.

scaling as $1/\sqrt{\omega_{\mathrm{H}}}$ with higher spectral weight by increasing pump field. In addition, the corresponding $\Delta_i(\omega)$ plotted in Supplementary Figs. 10(c)-(d) ($i = 1$) and Supplementary Figs. 10(e)-(f) ($i = 2$) have only one peak at frequency $\omega_{\mathrm{H,i}}$ due to uncoupled bands and the spectral weight of the peak is also monotonically increasing. The order parameter Δ_1 is stronger quenched compared to Δ_2 and $\omega_{\mathrm{H,1}}$ peak amplitude in $\Delta_1(\omega)$ spectra is larger than $\omega_{\mathrm{H,2}}$ peak amplitude in $\Delta_2(\omega)$ since the lower resonance Δ_1 experiences stronger excitation by the applied pump E^2 field.

The pump field dependence with $U = 0$ is further summarized in Supplementary Fig. 11 where (a) $\omega_{\mathrm{H,i}}$, (b) $\Delta_i(\omega_{\mathrm{H,i}})$, and (c) the spectral weight of Higgs mode $\omega_{\mathrm{H,i}}$ in the $\Delta E/E$ response, $\mathrm{SW}_{\mathrm{H},i}$, are shown as a function of pump field for Higgs mode $\omega_{\mathrm{H,1}}$ ($i = 1$, blue line) and Higgs mode $\omega_{\mathrm{H,2}}$ ($i = 2$, red line). In contrast to the result with finite interband interaction presented in Fig. 4 in the main text where the spectral weight of Higgs mode $\omega_{\mathrm{H,1}}$ grows at low pump fields before decreasing at elevated pump fluences, the spectral weight $\mathrm{SW}_{\mathrm{H},i}$ as well as the amplitude of the $\omega_{\mathrm{H,i}}$ peak in $\Delta_i(\omega)$ spectra increase monotonically up to a Δ_1 quench of roughly 90% with Higgs mode $\omega_{\mathrm{H,1}}$ dominating the coherent FWM response. A further increase of the pump field leads to a the complete quench of the order parameter Δ_1 and a decrease of the spectral weight of Higgs mode $\omega_{\mathrm{H,1}}$ to zero while $\mathrm{SW}_{\mathrm{H,2}}$ is further increasing. Here a transition from a damped oscillating Higgs phase ($\omega_{\mathrm{H,1}} > 0$, $\Delta_1(\omega_{\mathrm{H,1}}) > 0$, and $\mathrm{SW}_{\mathrm{H,1}} > 0$) to

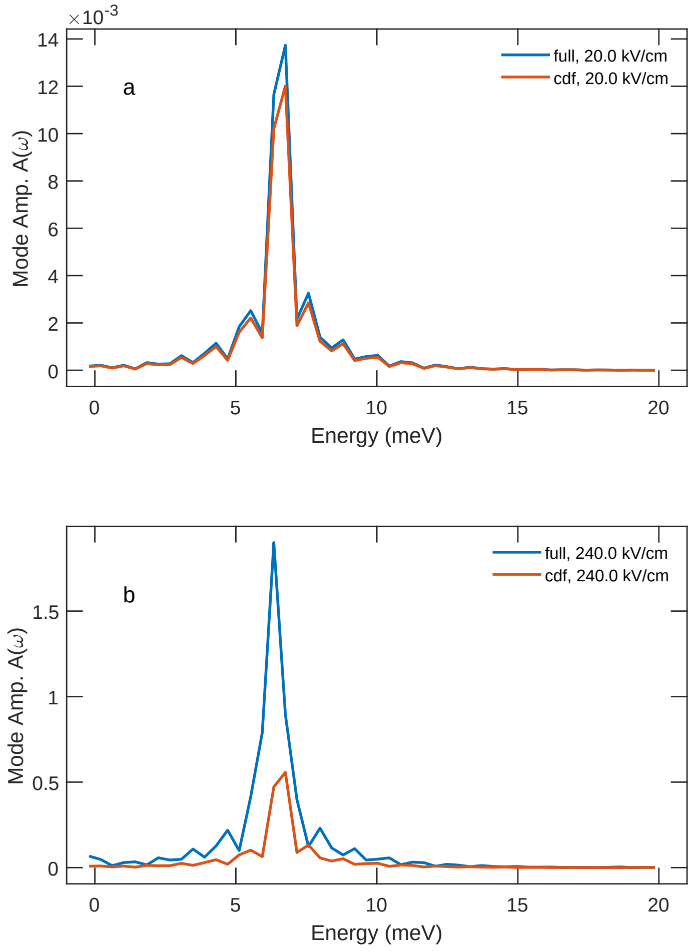

Supplementary Figure 12: Effect of charge-density fluctuations on coherent $\Delta E/E$ response. $\Delta E/E$ spectra for (**a**) a low pump field of 20 kV/cm and (**b**) a high pump field of 240 kV/cm. The full calculation (blue line) is compared with a calculation where light-induced collective effects are switched off (red line) such that the response is fully determined by charge-density fluctuations. While charge-density fluctuations dominate the coherent $\Delta E/E$ response at low pump fields, collective Higgs effects increase the nonlinear response by a factor of 4 at higher pump fields.

an exponentially decaying $\Delta_1(t)$ ($\omega_{\mathrm{H},1} = 0$ and $\Delta_1(\omega_{\mathrm{H},1}) = \mathrm{SW}_{\mathrm{H},1} = 0$) is observable. Note that the decrease of the spectral weight with interband coupling in Fig. 4 in the main text appears at a Δ_1 quench close to 15% while without interband coupling the decrease of spectral weight is only observable close to the complete quench ($\sim$ 90%) of the SC order parameter Δ_1. We conclude from this that the spectral weight decrease at the lower Higgs resonance mode with low quench is a direct consequence of spectral weight transfer between the Higgs modes and is induced by the large interband coupling between electron and hole pockets.

Supplementary Note 6.2: Effect of charge-density fluctuations on coherent FWM response

The detection of collective modes in BCS superconductors with linear spectroscopy is in general challenging, as they require a finite condensate momentum to couple to electromagnetic fields. The latter is obtained, for example, by dc supercurrent injection such that the Higgs mode can be detected with linear spectroscopy [27, 28]. The detection of the Higgs mode in the nonlinear response via third harmonic generation in ultrafast THz spectroscopy is also challenging

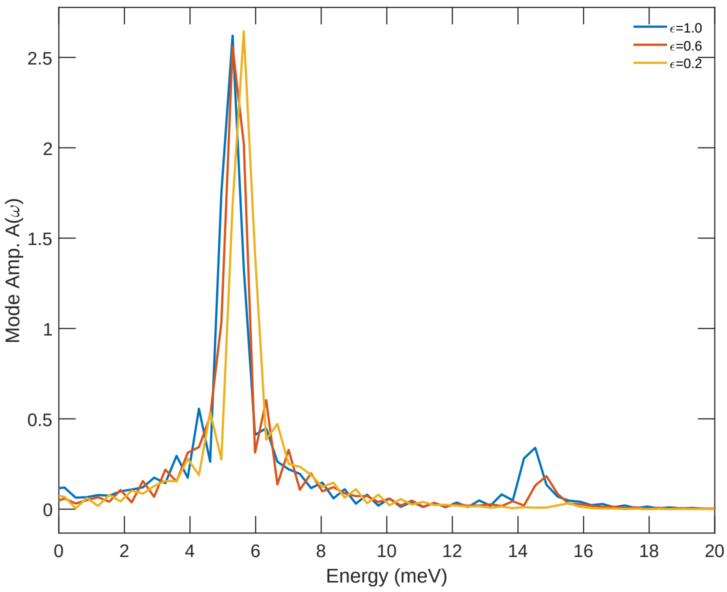

Supplementary Figure 13: Effect of particle-hole asymmetry on $\Delta E/E$ spectra. $\Delta E/E$ spectra for different e-pocket ellipticities show a decrease and broadening of $\omega_{\mathrm{H},2}$-peak with increasing ε while $\omega_{\mathrm{H},1}$-mode is not much affected by particle-hole asymmetry.

because charge-density fluctuations dominate over the Higgs mode within BCS theory in a clean system and in the perturbative excitation regime [29]. Recent studies have shown that Higgs modes can still be observed if electron-phonon coupling or impurities are considered [30, 31]. However, it has been demonstrated [17] that even in clean one-band BCS systems collective effects become important in the strong nonlinear non-perturbative regime, where the SC order parameter becomes quenched. The effect of charge-density fluctuations on the $\Delta E/E$ response in our studied multiband SC system here is illustrated in Supplementary Fig. 12, where calculated $\Delta E/E$ spectra are shown for a low (20 kV/cm, Supplementary Fig. 12(a)) and a high pump field (240 kV/cm, Supplementary Fig. 12(b)). The result of the full calculation (blue line) is compared with a calculation where light-induced changes in collective effects (red line) are switched off. The latter curve is obtained by replacing the time-dependent order parameter $\Delta(t)$ by the equilibrium SC gap Δ_0 on the right-hand side of the equation of motions (12). As a result, the fluctuations, $\delta\Delta(t) = \Delta(t) - \Delta_0$, of the SC order parameter are neglected, such that the calculated response is fully determined by charge-density fluctuations. The $\Delta E/E$ response is dominated by charge-density fluctuation at low pump fields, in agreement with earlier studies [29]. Here, the order parameters are close to their equilibrium values, such that this perturbative regime can be described by susceptibility expansions as before. The situation changes in the non-perturbative regime, where both gaps start to become quenched and the nonlinear response cannot be described by susceptibility expansions anymore. Here, collective effects dominate over charge-density fluctuations (Supplementary Fig. 12(b)) which is due to the different effect of the strong interband U and multi-pocket bandstructure on quasiparticles and Higgs collective modes in the non-perturbative excitation regime. We conclude from this that the coherent oscillations observed in the experiment at higher pump fluences result from the hybrid Higgs mode and not from charge-density fluctuations even within BCS theory in a clean system.

Supplementary Note 6.3: Hybrid Higgs mode in multi-band superconductors with particle-hole symmetry

As demonstrated in Ref. [32] the collective mode damping in the $\Delta E/E$ response depends on the details of the bandstructure. In particular, a large density of states (DOS) close to the Fermi surface yields stronger damping of the collective mode oscillations. This is different to the order parameter dynamics in an ideal system where the damping of the Higgs mode is independent of the bandstructure (the decay of the Higgs oscillations due to Landau

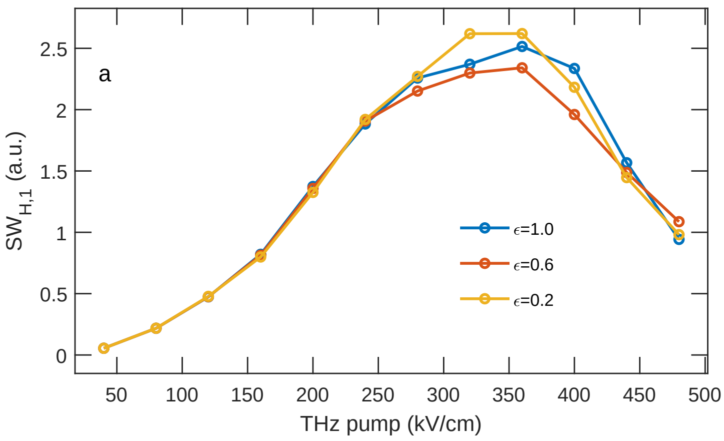

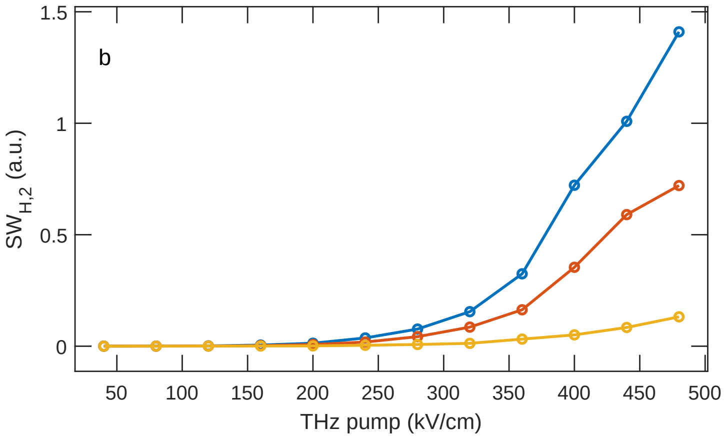

Supplementary Figure 14: Pump-field dependence of FWM spectral weight of (a) Higgs mode $\omega_{\mathrm{H},1}$ and (b) Higgs mode $\omega_{\mathrm{H},2}$ for different particle-hole asymmetries. Spectral weight of $\omega_{\mathrm{H},1}$-mode is not much affected by e-pocket ellipticity while spectral weight of mode $\omega_{\mathrm{H},2}$ decreases with growing e-pocket ellipticity.

damping in one-band superconductors is given by $t^{-1/2}$ while for a 2-band superconductor it is $t^{-3/2}$ as discussed in Ref. [33].) For a bandstructure with particle-hole symmetry, both modes experience the same damping in the $\Delta E/E$ response. However, the measured Fermi surface [21] exhibits particle-hole asymmetry in form of elliptical electron pockets which leads to different damping of the Higgs modes of electron and hole pockets in the $\Delta E/E$ response. To take this into account we have added particle-hole asymmetry using elliptical e-pockets consistent with the Femi-surface topology measured in Ref. [21]. The ellipticity of the e-pockets increases the DOS along pump field direction and thus increases the damping of the higher Higgs mode. The effect of particle-hole asymmetry is illustrated in Supplementary Fig. 13 where the $\Delta E/E$ spectra are shown for different ratios $\varepsilon = J_x/J_y$. The Higgs mode $\omega_{\mathrm{H},2}$ resonance in the $\Delta E/E$ spectra broadens and decreases with increasing ellipticity of the e-pocket due to increasing damping. The resulting pump-field dependence is studied in Supplementary Fig. 14 where the spectral weight of Higgs mode $\omega_{\mathrm{H},1}$ (Supplementary Fig. 13(a)) and $\omega_{\mathrm{H},2}$ (Supplementary Fig. 13(b)) are shown as a function of pump field. The spectral weight of Higgs mode $\omega_{\mathrm{H},2}$ decreases with increasing ellipticity of the electron pockets. Compared to that the pump-field dependence of the Higgs mode $\omega_{\mathrm{H},1}$ is not much affected by electron-hole asymmetry, i. e. the growing of $\mathrm{SW}_{\mathrm{H},1}$ at low pump fluence, followed by a saturation and then decrease at elevated E_{pump} is observable for all studied particle-hole asymmetries. This shows that the result of the pump-field dependence of $\omega_{\mathrm{H},1}$-mode is very robust since it does not depend on the details of the bandstructure. More specifically, the transfer of spectral weight between both Higgs modes observed via the specific pump-field dependence of $\omega_{\mathrm{H},1}$-mode is even visible when the $\omega_{\mathrm{H},2}$ peak is too small to be seen in the $\Delta E/E$ spectra. As a result, the particle-hole asymmetry between the electron and the hole pockets strongly suppresses the visibility of $\omega_{\mathrm{H},2}$ mode in the $\Delta E/E$ response which also complicates the experimental detection of the Higgs mode $\omega_{\mathrm{H},2}$ in addition to damping and limited detection bandwidth.

Supplementary Note 6.4: Origin of asymmetric Higgs mode lineshape

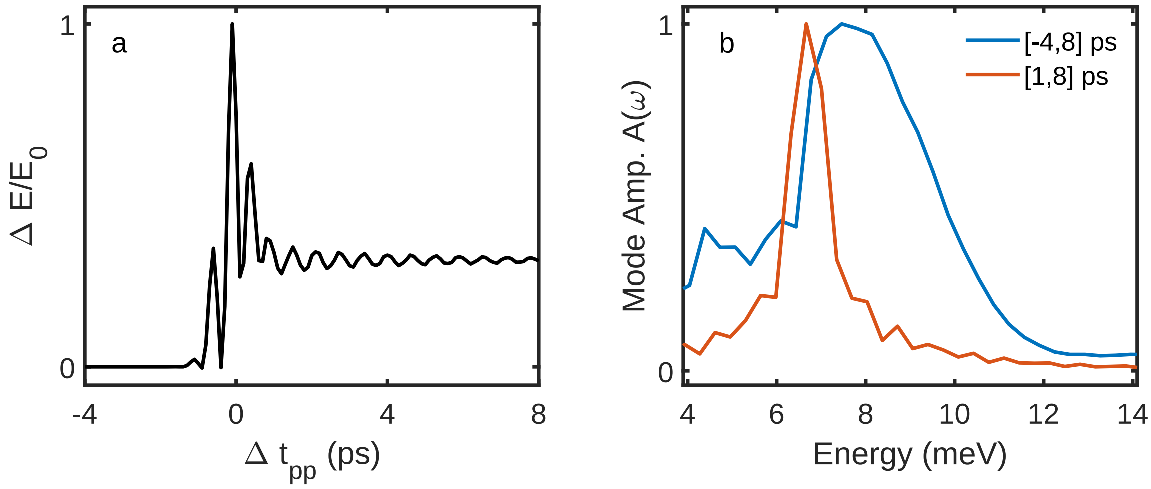

Supplementary Figure 15: Origin of asymmetric Higgs mode lineshape. **a**, Calculated dynamics of the differential transmission $\Delta E/E_0$ for $E_{\mathrm{pump}} = 225$ kV/cm. **b**, The corresponding calculated Higgs mode spectrum using a time window of $[-4, 8]$ ps (blue line) and $[1, 8]$ ps (red line) for the Fourier transformation.

Supplementary Fig. 15(a) shows the computed dynamics of the FWM signal $\Delta E/E_0$, calculated directly in the time domain for a pump field strength of $E_{\mathrm{pump}} = 225$ kV/cm. Note that we do not invoke any assumptions about how the experimentally measured multi-dimensional signal may connect to a nonlinear conductivity, but rather calculate what is measured directly in the time domain, from which a nonlinear conductivity can be extracted if needed. The corresponding calculated Higgs mode spectrum (b) is obtained by using a time window of $[-4, 8]$ ps (blue line) and $[1, 8]$ ps (red line) for the Fourier transformation. The latter has been used in Fig. 4 of the main text and only includes the time region after the THz-driven SC order parameter quench. Consequently, the spectrum only shows the collective mode signal, which yields a largely symmetric mode lineshape. Compared to that, the Fourier transform of the complete time trace also contains the APS nonlinear precession dynamics during the pulse, in addition to the collective mode dynamics after the pulse. As a result, the Higgs mode resonance overlaps with the second harmonic generation signal in the spectrum, resulting in a small shift of the resonance and an asymmetric mode lineshape, as observed in the experimental spectra such as Fig. 1e in the main text.

Supplementary References

[1] Lee, S. et al. Template engineering of Co-doped $BaFe_2As_2$ single-crystal thin films. *Nat. Mater.* **9**, 397 (2010).

[2] Kang, J. H. et al. Control of epitaxial $BaFe_2As_2$ atomic configurations with substrate surface terminations. *Nano Lett.* **18**, 6347-6352 (2018).

[3] Kang, J. H. et al. Superconductivity in Undoped $BaFe_2As_2$ by Tetrahedral Geometry Design. *Proc. Natl. Acad. Sci.* **117**, 21170 (2020).

[4] Kuehn, W., Reimann, K., Woerner, M. & Elsaesser, T. Phase-resolved two-dimensional spectroscopy based on collinear n-wave mixing in the ultrafast time domain. *J. Chem. Phys.* **130**, 164503 (2009).

[5] Maag, T. et al. Coherent cyclotron motion beyond Kohn's theorem. *Nat. Phys.* **12**, 119 (2016).

[6] Yang, X. et al. Terahertz-light quantum tuning of a metastable emergent phase hidden by superconductivity. *Nat. Mater.* **17**, 586 (2018).

[7] Woerner, M., Kuehn, W., Bowlan, P., Reimann, K.& Elsaesser, T. Ultrafast two-dimensional terahertz spectroscopy of elementary excitations in solids. *New J. Phys.* **15**, 025039 (2013).

[8] Ibrahim, A., Férachou, D., Sharma, G., Singh, K., Kirouac-Turmel, M. & Ozaki, T. Ultra-high dynamic range electro-optic sampling for detecting millimeter and sub-millimeter radiation. *Sci Rep* **6**, 23107 (2016).

[9] Hebling, J., Yeh, K.-L., Hoffmann, M. C., Bartal, B. & Nelson, K. A. Generation of high-power terahertz pulses by tilted-pulse-front excitation and their application possibilities. *J. Opt. Soc. Am. B* **25**, B6 (2008).

[10] Hirori, H., Doi, A., Blanchard, F. & Tanaka, K. Single-cycle terahertz pulses with amplitudes exceeding 1 MV/cm generated by optical rectification in $LiNbO_3$. *Appl. Phys. Lett.* **98**, 091106 (2011).
[11] Yang, X., Vaswani, C., Sundahl, C., Mootz, M., Luo, L., Kang, J., Perakis, I., Eom, C. & Wang, J. Lightwave-driven gapless superconductivity and forbidden quantum beats by terahertz symmetry breaking. *Nat. Photon.* **13**, 707 (2019).
[12] Yang, X. et al. Ultrafast nonthermal terahertz electrodynamics and possible quantum energy transfer in the Nb_3Sn superconductor. *Phys. Rev. B* **99**, 094504 (2019).
[13] Tu, J. J. et al. Optical properties of the iron arsenic superconductor $BaFe_{1.85}Co_{0.15}As_2$. *Phys. Rev. B.* **82**, 174509 (2010).
[14] Charnukha, A. Optical conductivity of iron-based superconductors. *J. Phys.: Condens. Matter.* **26**, 253203 (2014).
[15] Yang, X. et al. Nonequilibrium pair breaking in $Ba(Fe_{1-x}Co_x)_2As_2$ superconductors: evidence for formation of a photoinduced excitonic state. *Phys. Rev. Lett.* **121**, 267001 (2018).
[16] Vaswani, C. et al. Discovery of Terahertz Second Harmonic Generation from Lightwave Acceleration of Symmetry-Breaking Nonlinear Supercurrents. *Phys. Rev. Lett.* **124**, 207003 (2020).
[17] Mootz, M., Wang, J. & Perakis, I. E. Lightwave Terahertz Quantum Manipulation of Non-equilibrium Superconductor Phases and their Collective Modes. *Phys. Rev. B* **102**, 054517 (2020).
[18] Stephen, M. J. Transport Equations for Superconductors. *Phys. Rev.* **139**, A197 (1965).
[19] Yang, F. & Wu, M. W. Gauge-invariant microscopic kinetic theory of superconductivity: Application to the optical response of Nambu-Goldstone and Higgs modes. *Phys. Rev. B* **100**, 104513 (2019).
[20] Nambu, Y. Quasi-Particles and Gauge Invariance in the Theory of Superconductivity. *Phys. Rev.* **117**, 648 (1960).
[21] Liu, C., Kondo, T., Fernandes, R. M. et al. Evidence for a Lifshitz transition in electron-doped iron arsenic superconductors at the onset of superconductivity. *Nature Phys.* **6**, 419-423 (2010).
[22] Fernandes, R. M. & Schmalian, J. Competing order and nature of the pairing state in the iron pnictides. *Phys. Rev. B*, **82**, 014521 (2010).
[23] Murotani, Y., Tsuji, N. & Aoki, H. Theory of light-induced resonances with collective Higgs and Leggett modes in multiband superconductors. *Phys. Rev. B*, **95**, 104503 (2017).
[24] Yang, X., Luo, L., Mootz, M., Patz, A., Bud'ko, S. L., Canfield, P. C., Perakis, I. E. & Wang, J. Nonequilibrium Pair Breaking in $Ba(Fe_{1-x}Co_x)_2As_2$ Superconductors: Evidence for Formation of a Photoinduced Excitonic State. *Phys. Rev. Lett.* **121**, 267001 (2018).
[25] Vorontsov, A. B., Vavilov, M. G. & Chubukov, A. V. Superconductivity and spin-density waves in multiband metals. *Phys. Rev. B* **81**, 174538 (2010)
[26] Yuzbashyan, E. A. & Dzero, M. Dynamical Vanishing of the Order Parameter in a Fermionic Condensate. *Phys. Rev. Lett.* **96**, 230404 (2006).
[27] Nakamura, S., Iida, Y., Murotani, Y., Matsunaga, R., Terai, H.& Shimano, R. Infrared Activation of the Higgs Mode by Supercurrent Injection in Superconducting NbN. *Phys. Rev. Lett.* **122**, 257001 (2019).
[28] Moor, A., Volkov, A. F. & Efetov, K. B. Amplitude Higgs Mode and Admittance in Superconductors with a Moving Condensate. *Phys. Rev. Lett.* **118**, 047001 (2017).
[29] Cea, T., Castellani, C. & Benfatto, L. Nonlinear optical effects and third-harmonic generation in superconductors: Cooper pairs versus Higgs mode contribution. *Phys. Rev. B* **93**, 180507 (2016).
[30] Cea, T., Barone, P., Castellani, C. & Benfatto, L. Polarization dependence of the third-harmonic generation in multiband superconductors. *Phys. Rev. B* **97**, 094516 (2018).
[31] Murotani, Y. & Shimano, R. Nonlinear optical response of collective modes in multiband superconductors assisted by nonmagnetic impurities. *Phys. Rev. B* **99**, 224510 (2019).
[32] Udina, M., Cea, T. & Benfatto, L. Theory of coherent-oscillations generation in terahertz pump-probe spectroscopy: From phonons to electronic collective modes. *Phys. Rev. B* **100**, 165131 (2019).
[33] Cui, T., Schütt, M., Orth, P. P. & Fernandes, R. M. Postquench gap dynamics of two-band superconductors. *Phys. Rev. B* **100**, 144513 (2019).
